# Zinc silicate microspheres-mediated bacterial metabolic interference and microenvironment remodeling for chronic wound repair

**DOI:** 10.1016/j.bioactmat.2026.07.052

**Published:** 2026-09-17

**Authors:** Fanyan Deng, Zizhuo Liu, Yiran Shao, Xiude Chen, Kexin Zhang, Yanping Feng, Jinyu Zeng, Dong Han, Shunxiang Xu, Congqin Ning

**Affiliations:** a The Education Ministry Key Lab of Resource Chemistry and Shanghai Frontiers Science Center of Biomimetic Catalysis and Shanghai Engineering Research Center of Green Energy Chemical Engineering, Shanghai Normal University, Shanghai, PR China; b Department of Orthopaedics and Traumatology, Faculty of Medicine, The Chinese University of Hong Kong, Hong Kong SAR, PR China; c Department of Plastic and Reconstructive Surgery, Shanghai Ninth People's Hospital, Shanghai Jiao Tong University School of Medicine, Shanghai, PR China; d SHNU-YAPENG Joint Lab of Tissue Repair Materials, Shanghai Yapeng Biological Technology Co., Ltd, Shanghai, PR China

**Keywords:** zinc silicate microspheres, Chronic wound, Antibacterial, Metabolic interference, Microenvironment remodel

## Abstract

Chronic wounds have presented a significant clinical challenge due to persistent infection, uncontrolled inflammation, and impaired tissue regeneration. Existing therapeutic platforms have failed to integrate these critical aspects into a single, cohesive strategy. To address this, a novel multifaceted agent, zinc silicate flower-like microsphere (ZnSi FMPs), was designed with controlled hierarchical architecture to achieve chronic wound repair by mediating bacterial metabolic interference and microenvironment remodeling. The unique nanosheet structure with sustained Zn^2+^ release endows ZnSi FMPs with favorable pH-independent antibacterial activity. This activity is mechanistically rooted in metabolic interference against *Staphylococcus aureus* (*S*. *aureus*), including disruption of *ABC transporters* and inhibition of the biosynthesis of essential amino acids (*valine, leucine, isoleucine*) and *ribosomes*. Concurrently, ZnSi FMPs can promote microenvironment remodeling by modulating inflammatory responses and angiogenesis, evidenced by suppressed inflammation mediated by the *complement and coagulation cascades pathway* and robust activation of the *Areg/HB-EGF*-mediated *ErbB signaling pathway*. Hence, in both *S. aureus*-infected and diabetic wound models (mice and minipigs), ZnSi FMPs exhibited superior chronic wound healing capacity compared with commercial silver sulfadiazine® (AgSD) and 45S5 bioglass®. This improved healing was achieved through enhanced antibacterial activity, anti-inflammatory effects, and angiogenesis, accompanied by elevated expression of *CD163, CD206, CD31* and *α-SMA*. By synergizing bacterial metabolic interference with microenvironment regulation, ZnSi FMPs effectively facilitate tissue healing and provide a promising candidate for clinical wound treatment.

## Highlights

• Hierarchical zinc silicate flower-like microspheres (ZnSi FMPs) are successfully synthesized for chronic wound repair via bacterial metabolic interference and wound microenvironment remodeling.
• ZnSi FMPs interfere with the metabolism of *S. aureus* by disrupting *ABC transporters* and inhibiting branched-chain amino acid (*valine, leucine, isoleucine*) and *ribosome* biosynthesis, thus realizing pH-independent antibacterial efficacy.
• ZnSi FMPs effectively remodel wound microenvironment by suppressing inflammation driven by the *complement and coagulation cascades pathway* and activating *Areg*/*HB-EGF*-mediated *ErbB signaling pathway* to facilitate tissue regeneration.
• ZnSi FMPs exhibit better wound healing efficacy in mice and minipig chronic wound models, outperforming commercial silver sulfadiazine and 45S5 bioglass.

## Graphical abstract

**Figure:**
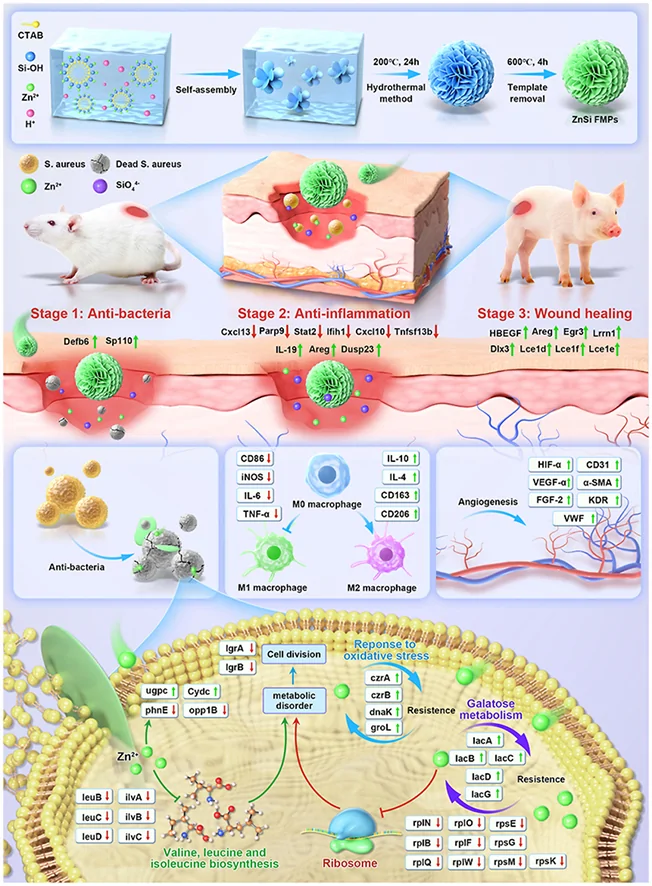

## 1 Introduction

Chronic wounds, such as infected wounds, diabetic wounds and pressure sores, have posed significant clinical challenges due to prolonged inflammation, bacterial colonization, and impaired angiogenesis [1]. Globally, the prevalence of chronic wounds has reached 1.67%, which means more than 130 million individuals have been troubled by chronic wounds [2]. Nevertheless, according to the report of “Global Wound Care Market” (2024), the value of the wound care market has reached to $21.11 billion in 2024, and is expected to reach $30.27 billion by 2031 [3]. In addition, the pH of natural skin is acidic, typically ranging from 4.50 to 6.50, whereas chronic wounds present an elevated pH range of 7.15 to 8.90, which facilitates bacterial proliferation and inflammation, further preventing wound healing [4]. Consequently, the effective treatment of chronic wounds has remained a pressing challenge.

Antibiotics and growth factors have been used in clinical settings to treat the issues of infection and impaired tissue regeneration in the past two decades [5]. However, continuous antibiotic therapy has not only contributed to bacterial resistance, but recurrent infections caused by resistant strains have further inhibited wound healing, and even promoted wound deterioration [6]. Besides, the high cost of endogenous growth factors and increased risk of immune response, inflammation and cancer also have restricted the application of growth factors [7]. Hence, the research and development of multifunctional biomaterials capable of simultaneously inhibiting infection and facilitating tissue repair has maintained a highly desirable and valuable direction.

Given the increasing prevalence of multidrug-resistant pathogens and the limitations associated with antibiotic use, metal-based materials with good antibacterial activity have been reported for wound healing, including silver (Ag), gold (Au), copper (Cu), zinc (Zn), cerium (Ce), titanium (Ti) and yttrium (Y) etc., with the form of metals, metal oxide particles and nanoenzymes [8]. These metal elements have exhibited broad-spectrum antibacterial properties by generating reactive oxygen species (ROS), disrupting bacterial cell membranes, or interfering with essential metabolic processes [9]. In particular, metal-based materials, have been widely applied in the wound care market, such as silver sulphadiazine (AgSD) powder, Acticoat™, Silvasorb® and zinc oxide (ZnO) oil agent and cream [10]. Nevertheless, Ag-based materials have faced the drawbacks of cytotoxicity and limited biocompatibility, which restrict their application [11].

Zn, as an essential trace element, plays a pivotal role in numerous physiological processes, including enzymatic activity, immune function, tissue repair and angiogenesis [12]. Several studies have reported that Zn deficiency is associated with developmental abnormalities, impaired immune responses, delayed wound healing, and metabolic disorders such as diabetes-related microvascular complications [13]. In wound repair, apart from the excellent antibacterial property, results have proven that the addition of Zn can accelerates collagen synthesis and epithelialization by promoting the proliferation of fibroblast and stabilizing collagen fibrils [14]. Clinical studies on burn patients have demonstrated that topical Zn formulations can enhance epithelial migration and shorten healing time [15]. Moreover, Zn-loaded dressing has been reported to modulate the immune microenvironment by promoting macrophage polarization toward M2 phenotype, which is crucial for reducing chronic inflammation in diabetic wounds [16].

In addition to metal-based materials, bioactive inorganic particles have also attracted significant attention in the field of wound healing, considering its good biocompatibility and promotion effect on tissue regeneration. To date, phosphate-, silicate- and borate-based bioceramics have been widely studied for wound healing [17,18]. Moreover, several products incorporating bioactive inorganic minerals have been used in wound repair, such as Novamin®, Vitoss®, DermFactor and 45S5 bioglasses® [19]. A common feature among these bioactive inorganic minerals is the presence of silicon (Si) elements, highlighting the notable efficacy of Si in skin wound healing. Several studies have demonstrated that the addition of Si element can significantly accelerate wound healing by promoting the expression of EGF to achieve anti-inflammatory and promoting the secretion of healing factors of ANG1, bFGF, and VEGF [17,20]. Since the similarity between Ca-P related bioceramics and natural bone components, and the widely application of Ca-P related bioceramics in bone repair have also raised the concern of possible ectopic ossification in skin wound healing [21]. Given the superiority of Si in promoting wound healing, developing a Si-containing alternative to conventional Ca-P based bioceramics is feasible with broad prospect.

**Scheme 1:**
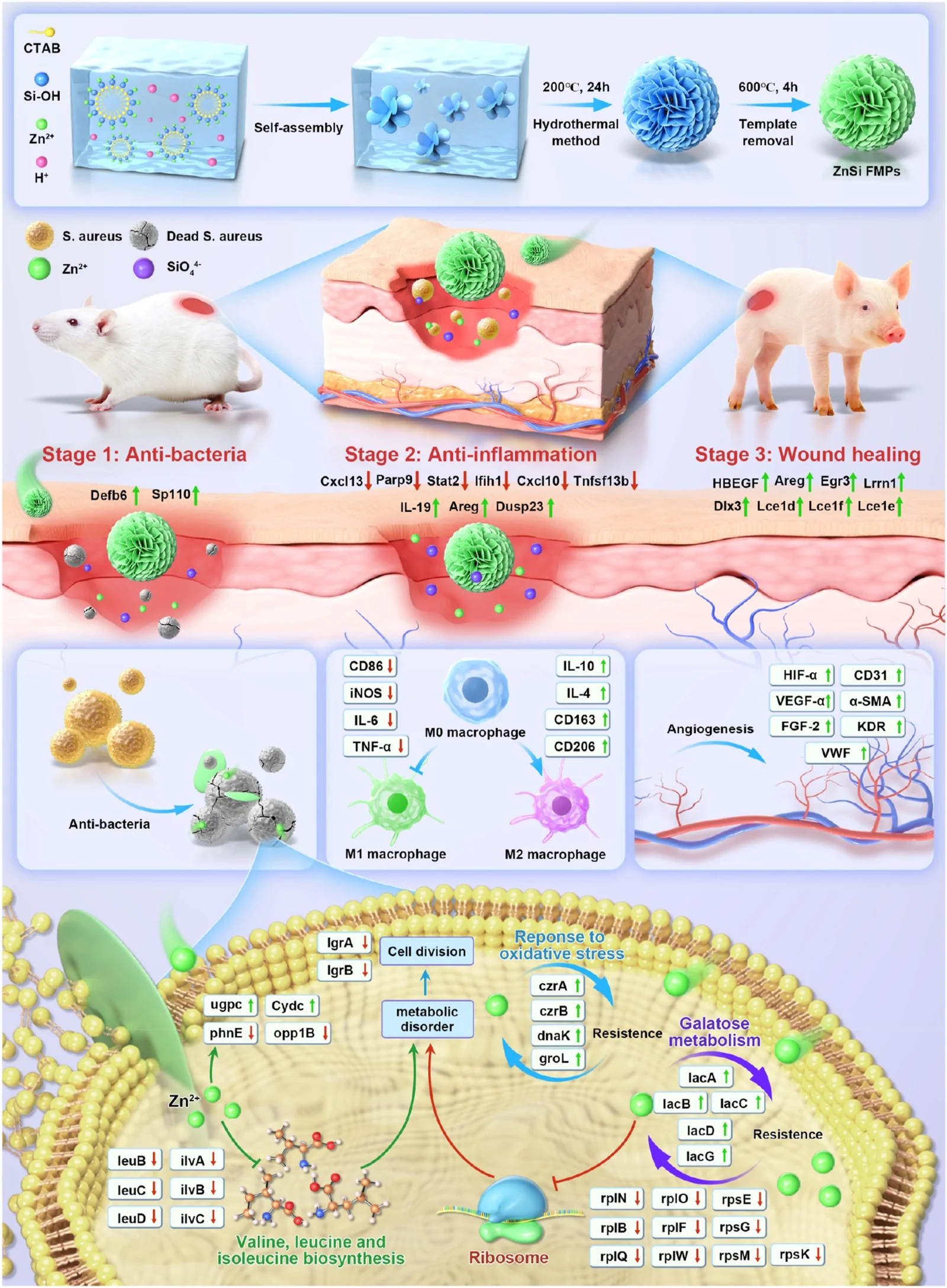
The process of materials preparation and mechanism of ZnSi FMPs for infected-wound healing.

Zinc silicate (Zn_2_SiO_4_, noted as ZnSi), has been extensively utilized in the field of coatings, luminescent and catalysis [22]. In recent years, the bioactivity of ZnSi in tissue repair has been increasingly recognized, particularly its therapeutic potential in wound tissue [23]. However, existing relevant studies mainly focus on the biological activities induced by released zinc and silicate ions, while neglecting the morphological modulation of zinc silicate as well as the correlation between material microstructure and biological performance. To fill this research gap, ZnSi flower-like microspheres (ZnSi FMPs) assembled from nanosheets were designed and applied for chronic wound healing as shown in Scheme 1. Following the systematic optimization of synthetic parameters, including the synthetic factors of raw materials, CTAB content, reaction time and temperature, we investigated the integrated therapeutic potential of ZnSi FMPs both *in vitro* and *in vivo*. Specifically, this study explored how the unique nanosheet architecture and bioactive Zn^2+^ release execute a metabolic interference against *S. aureus*. Beyond antibacterial efficacy, we evaluated the capacity and related mechanism of ZnSi FMPs on regulating remodel microenvironment including anti-inflammation and angiogenesis. To validate clinical translatability, chronic wound models including *S. aureus*-infected and diabetic wounds were established in both mice and minipigs. The therapeutic performance of ZnSi FMPs was compared with commercial silver sulfadiazine (AgSD) and 45S5 bioglass, to demonstrate its superiority in achieving chronic tissue regeneration.

## 2 Results

### 2.1 Synthesis and characterization of ZnSi FMPs

**Fig. 1:**
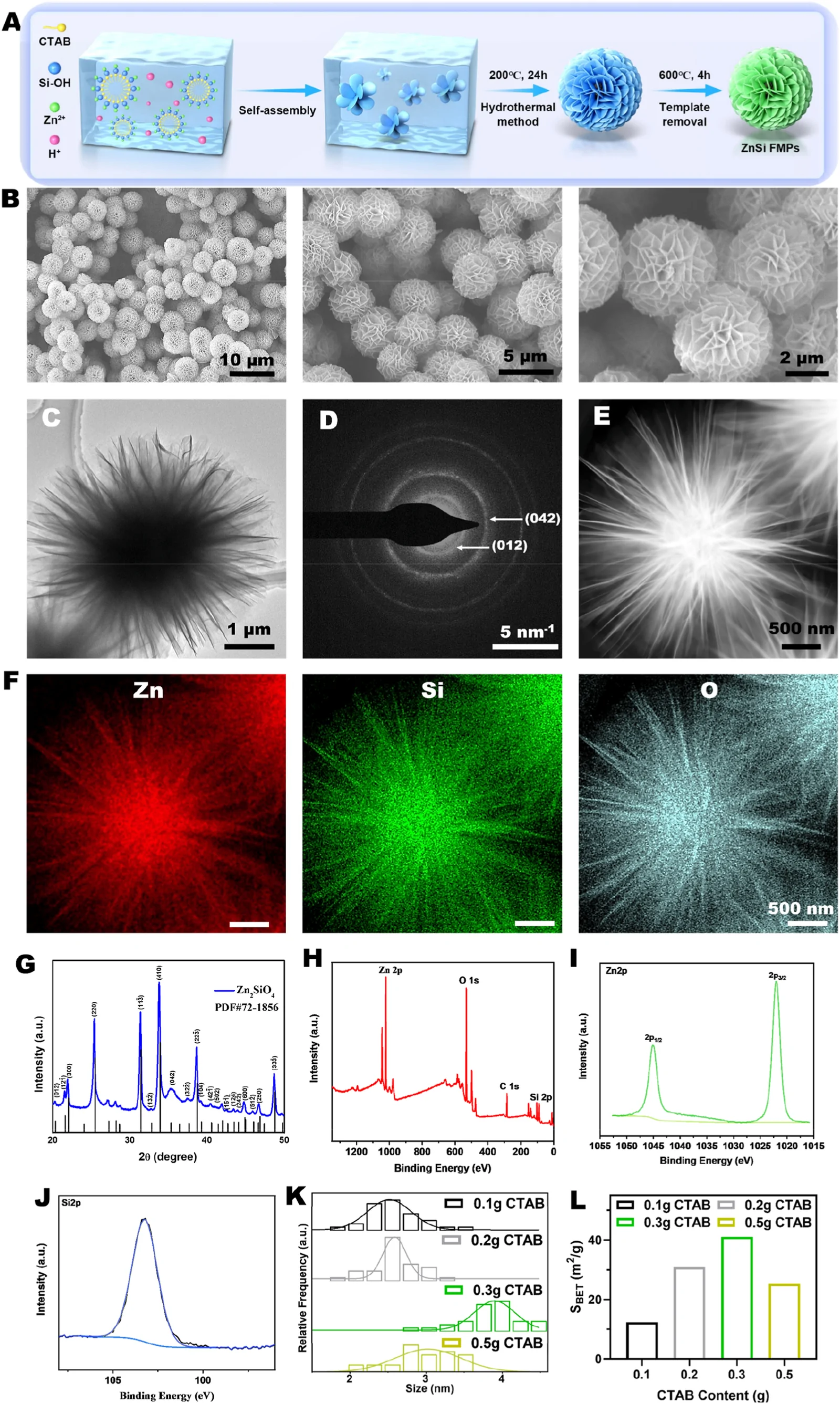
Characterization of Zn_2_SiO_4_ flower-like microspheres (ZnSi FMPs). (A) Schematic illustration of the synthetic method for ZnSi FMPs. (B) SEM with different magnification, (C) TEM, (D) SAED pattern, (E, F) Elements mapping images, (G) XRD and (H-J) XPS patterns of ZnSi FMPs. (K) Particle size distribution and (L) Specific surface areas of ZnSi FMPs synthesized under different content of CTAB.

ZnSi FMPs were synthesized through a hydrothermal method of the surfactant CTAB, microsphere precursor TEOS and Zn(NO_3_)_2_, and nitric acid modulator in an aqueous solution at 200°C as shown in Fig. 1A. The scanning electron microscopy (SEM) results as shown in Fig. 1B demonstrated a well-assembled spherical morphology of ZnSi FMPs with an average diameter of ∼3.8 μm, and the higher magnification further demonstrated that ZnSi FMPs were composed of densely packed nanosheets, forming a three-dimensional (3D) hierarchical architecture. Structural characterization of ZnSi FMPs was also performed using transmission electron microscopy (TEM) as shown in Fig. 1C, flower-like nanosheets growing outward from the central core, and the sheet-stacked pores could be clearly observed. The corresponding elements mapping images as shown in Fig. 1E and F revealed that Zn, Si and O elements were uniformly distributed in the ZnSi FMPs. X-ray diffraction (XRD) patterns of the as-synthesized microspheres as shown in Fig. 1G matched well with PDF#72-1856 (Zn_2_SiO_4_), indicating single phase of hexagonal Zn_2_SiO_4_ powders were obtained. Moreover, the phase composition of ZnSi FMPs was further confirmed by selected area electron diffraction (SAED) pattern as shown in Fig. 1D, the results appeared polycrystalline diffraction rings, which is consistent with the presence of multiple randomly oriented nanosheets within the microsphere. Fig. 1H–J showed the XPS spectra of ZnSi FMPs, the full survey spectrum in Fig. 1H confirmed the presence of Zn, Si and O elements. The high-resolution Zn 2p spectrum in Fig. 1I presented two split peaks at 1022.0 eV (Zn 2p_3_/_2_) and 1045.1 eV (Zn 2p_1_/_2_), which were characteristic signals of Zn^2+^ in zinc silicate. The Si 2p peak in Fig. 1J was located at the binding energy corresponding to Si^4+^ of Zn_2_SiO_4_.

The synthetic mechanism of ZnSi FMPs was subsequently investigated in depth, the synthetic factors of raw materials, CTAB content, reaction time and temperature as shown in Table S1 were explored and the results were shown in Fig. S1. ZnF_2_, ZnCl_2_, ZnSO_4_•7H_2_O, Zn(NO_3_)_2_•6H_2_O, Zn(CH_3_COO)_2_, Na_2_SiO_3_•9H_2_O and TEOS were chosen to investigate the effect of raw materials on morphology of ZnSi FMPs as shown in Fig. S1A. Obviously, among the various zinc precursors evaluated, only Zn(NO_3_)_2_•6H_2_O and Zn(CH_3_COO)_2_ enabled the formation of microspherical structures, but the resulting particles of Zn(CH_3_COO)_2_ exhibited incomplete crystallization, and TEOS was identified to be the only suitable source of Si. The influence of CTAB content on ZnSi FMPs formation was systematically investigated as shown in Fig. S1B and S1C, it was found most of the products consisted predominantly of amorphous nanoparticles with only trace amounts of ZnSi FMPs emerging from the solid matrix when CTAB content was 0 g, indicating that crystallization is essential for microsphere formation. When 0.05 g of CTAB was introduced, the yield of ZnSi FMPs significantly increased, demonstrating that CTAB can significantly promote the crystallization process. At 0.1 g CTAB, complete transformation of the solid matrix into crystalline ZnSi FMPs was achieved. However, the nanosheet assembly was relatively loose, indicating CTAB also has an effect on assembly of microspheres. Further increasing the CTAB content from 0.1 g to 0.5 g led to pronounced changes in both size and assembly morphology. Fig. 1K and L presented the change of particle size and specific surface area with the increase of CTAB content, the average particle size of ZnSi FMPs increased from 2.6 μm to 3.8 μm and then decreased to 3.0 μm, while the specific surface area rose from 12.4 m^2^/g to 41.0 m^2^/g before declining to 25.3 m^2^/g as CTAB content increased from 0.1 g to 0.3 g and then to 0.5 g. Pore size distribution and pore volume as shown in Fig. S2A and S2B showed ZnSi FMPs possessed typical mesoporous structures, and ZnSi FMPs with 0.3 g CTAB had the largest total pore volume. These trends indicated that an optimal CTAB concentration could facilitate the ordered assembly of nanosheets into larger, more porous microspheres—maximizing both size and surface area. Based on these results, 0.3 g of CTAB was determined to be optimal for the synthesis of well-defined ZnSi FMPs.

Finally, the reaction time and temperature were also explored as shown in Fig. S1D and S1E, after 6 h of hydrothermal treatment, only amorphous nanoparticles were observed. At 8 h, early-stage microsphere formation was detected, and isolated fully formed ZnSi FMPs appeared after 12 h. Completely assembled and uniformly sized ZnSi FMPs obtained with the reaction time increased to 24 h, indicating sufficient reaction time is critical for structural maturation of ZnSi FMPs. Similarly, a sufficiently high reaction temperature was also demanded for the formation of ZnSi FMPs, the products of 150°C were amorphous nanoparticles, and stacked ZnSi FMPs with a small number of amorphous nanoparticles could be observed at 180°C, whereas hydrothermal at 200°C was required to obtain fully crystalline and morphologically uniform ZnSi FMPs.

Moreover, Fig. S3 showed the morphological change, pH variation, and ion release profiles of the ZnSi FMPs during immersion over 21 days. As displayed in the SEM micrographs of Fig. S3A, no obvious morphological differences were observed at day 0, 1, 4, 7, 14 and 21, the samples remained intact without obvious collapse, demonstrating the favorable structural stability of ZnSi FMPs in aqueous environment. Meanwhile, the pH value in Fig. S3B fluctuated only within 7.30-7.40 over 21 days, which provided a favorable local pH microenvironment. Fig. S3C and S3D illustrated the cumulative release profiles of Zn and Si ions, respectively. Both Zn and Si ions exhibited continuous, sustained release trends from day 1 to day 21. At day 21, the cumulative released concentration of Zn ions reached 0.85 mg/L, while the cumulative concentration of Si ions reached 13.69 mg/L.

### 2.2 pH-independent antibacterial activity of ZnSi FMPs *in vitro*

**Fig. 2:**
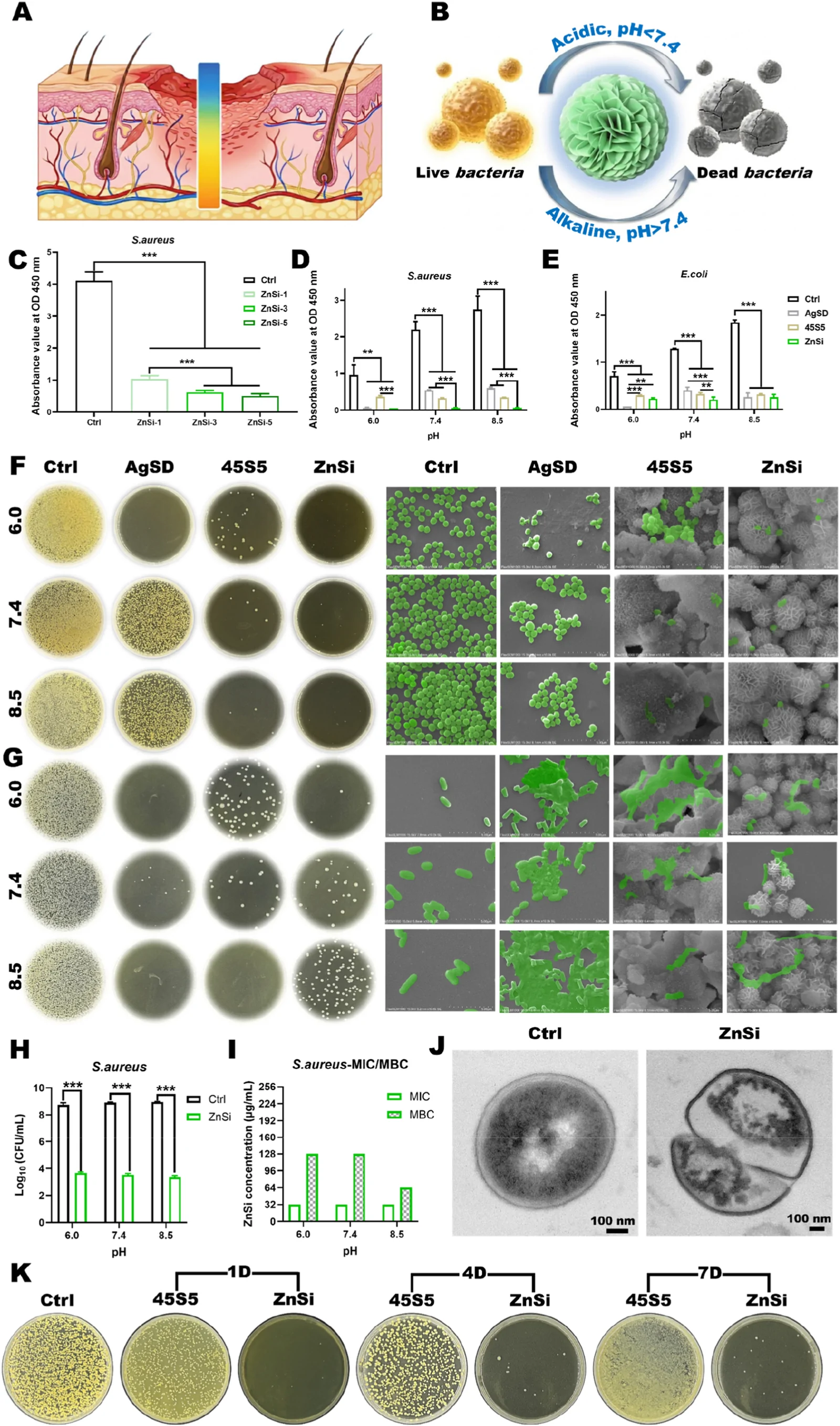
The *in vitro* antibacterial activity of ZnSi FMPs. Scheme of (A) normal skin and chronic wound and (B) the pH-independent antibacterial effect of ZnSi FMPs. (C) Absorbance results of *S. aureus* after treatment with different ZnSi content (n = 3). ***p < 0.001, by one-way ANOVA with Bonferroni *post hoc* test. Absorbance results of (D) *S. aureus* and (E) *E. coli* after treatment with Ctrl, AgSD, 45S5 and ZnSi at pH 6.0, 7.4 and 8.5 (n = 3). **p < 0.01,***p < 0.001, by two-way ANOVA with Bonferroni *post hoc* test. Agar plate results and SEM morphology of (F) *S. aureus* and (G) *E. coli* after treatment with samples. (H) Quantitative colony counting (log_10_ CFU) (n = 3). ***p < 0.001, by two-way ANOVA with Bonferroni *post hoc* test, and (I) MIC/MBC (μg/mL) of *S. aureus* after treatment with ZnSi FMPs at pH 6.0, 7.4 and 8.5 (n = 3). (J) TEM morphology of *S. aureus* after treatment with ZnSi FMPs. (K) Agar plate experimental results of *S. aureus* cultured with Ctrl, 45S5 and ZnSi groups after 1D, 4D and 7D degradation at pH = 7.4.

It is known that pH plays an important role in wound healing. As shown in Fig. 2A, studies have found that normal skin pH ranges from 4.50 to 6.50, while chronic wound shows an elevated pH of 7.15 to 8.90 [24]. The antibacterial effect of ZnSi FMPs was investigated and shown in Fig. 2. ZnSi FMPs exhibited outstanding antibacterial efficacy in a dose-dependent manner as shown in Fig. 2C, when tested against *S. aureus* with an initial bacterial of 1 × 10^7^ CFU/mL, ZnSi-1, ZnSi-3, and ZnSi-5 (1, 3, 5 mg ZnSi FPMPs, abbreviated as ZnSi-1, ZnSi-3 and ZnSi-5) achieved antibacterial rates exceeding 70% after 24 h, with statistically significant differences (P < 0.001). Notably. ZnSi-3 and ZnSi-5 achieved high inhibition rates of 85.0% and 87.8%, respectively, which were obviously higher than that of ZnSi-1, and no significant difference was found between ZnSi-3 and ZnSi-5. Thus, ZnSi-3 was selected for subsequent experiments. Phosphate Buffered Saline (PBS) with different pH values (6.0, 7.4 and 8.5) was used to imitate the variable pH environments of the acidic natural skin and alkaline chronic wound, and AgSD and 45S5 were used as control groups. The absorbance results, agar plate results and SEM morphologies of *S. aureus* and *E. coli* after treatments were shown in Fig. 2D–G., when tested against the initial bacterial concentration of 1 × 10^6^ CFU/mL, the bacterial activity of the Ctrl group obviously enhanced with the increase of pH value, confirming that the alkaline environment is favorable for bacterial growth. All experimental groups could suppress the growth of *S. aureus* and *E. coli*, with significant differences (**, P < 0.01). These findings were corroborated by agar plate assays and SEM images as shown in Fig. 2F and G. In the Ctrl group, bacteria displayed intact and smooth morphologies. Conversely, wrinkled dead bacteria could be observed in all experimental groups of AgSD, 45S5 and ZnSi. Especially, ZnSi FMPs exhibited stronger antibacterial potency against *S. aureus* than *E. coli* under same treatment conditions. Moreover, ZnSi FMPs demonstrated a strong and pH-independent antibacterial performance against *S. aureus* across all tested conditions, outperforming both AgSD and 45S5, indicating its outstanding antibacterial property.

To further evaluate the pH-independent antibacterial performance of ZnSi FMPs, the bacterial colony counting, minimum inhibitory concentration (MIC, μg/mL) and minimum bactericidal concentration (MBC, μg/mL) of ZnSi against *S. aureus* at pH 6.0, 7.4 and 8.5 were performed and presented in Fig. 2H and I. Across all three pH values, the Ctrl groups maintained a high bacterial viability at approximately log_10_ 8.9 CFU/mL. After incubation with ZnSi FMPs, the viable count of *S. aureus* reduced to ∼log_10_ 3.5 CFU/mL at every tested pH, with significant differences (***, P < 0.001). This outcome confirmed the robust bactericidal activity of ZnSi against *S. aureus* across acidic, neutral and weakly alkaline conditions. Fig. 2I further quantified the MIC of ZnSi against *S. aureus* remained constant at 32 μg/mL regardless of ambient pH, and the corresponding MBC values were 128, 128 and 64 μg/mL. Collectively, these results all demonstrated the outstanding pH-independent antibacterial performance of ZnSi FMPs. Moreover, TEM images as shown in Fig. 2J visualized the ultrastructural damage of *S. aureus* induced by ZnSi FMPs. The Ctrl group possessed complete spherical cells with intact membranes and homogeneous cytoplasm, while ZnSi-treated *S. aureus* showed collapsed cell envelopes, deformed morphology and massive cytoplasmic leakage, demonstrating that ZnSi destroys bacterial membrane integrity to kill *S. aureus*.

The long-term antibacterial durability of ZnSi FMPs was further evaluated after degradation for 1, 4, and 7 days as shown in Fig. S2 and Fig. 2K. 45S5 showed a marked decline in antibacterial activity over time, while ZnSi FMPs maintained robust performance, the antibacterial rate of ZnSi FMPs still reached 95.0% after 1D degradation and remained as high as 87.0% after 7D degradation. This prolonged efficacy highlighted the potential of ZnSi FMPs for managing chronic wounds, where long-term infection control is critical.

### 2.3 Investigation into the antibacterial mechanism of ZnSi FMPs

**Fig. 3:**
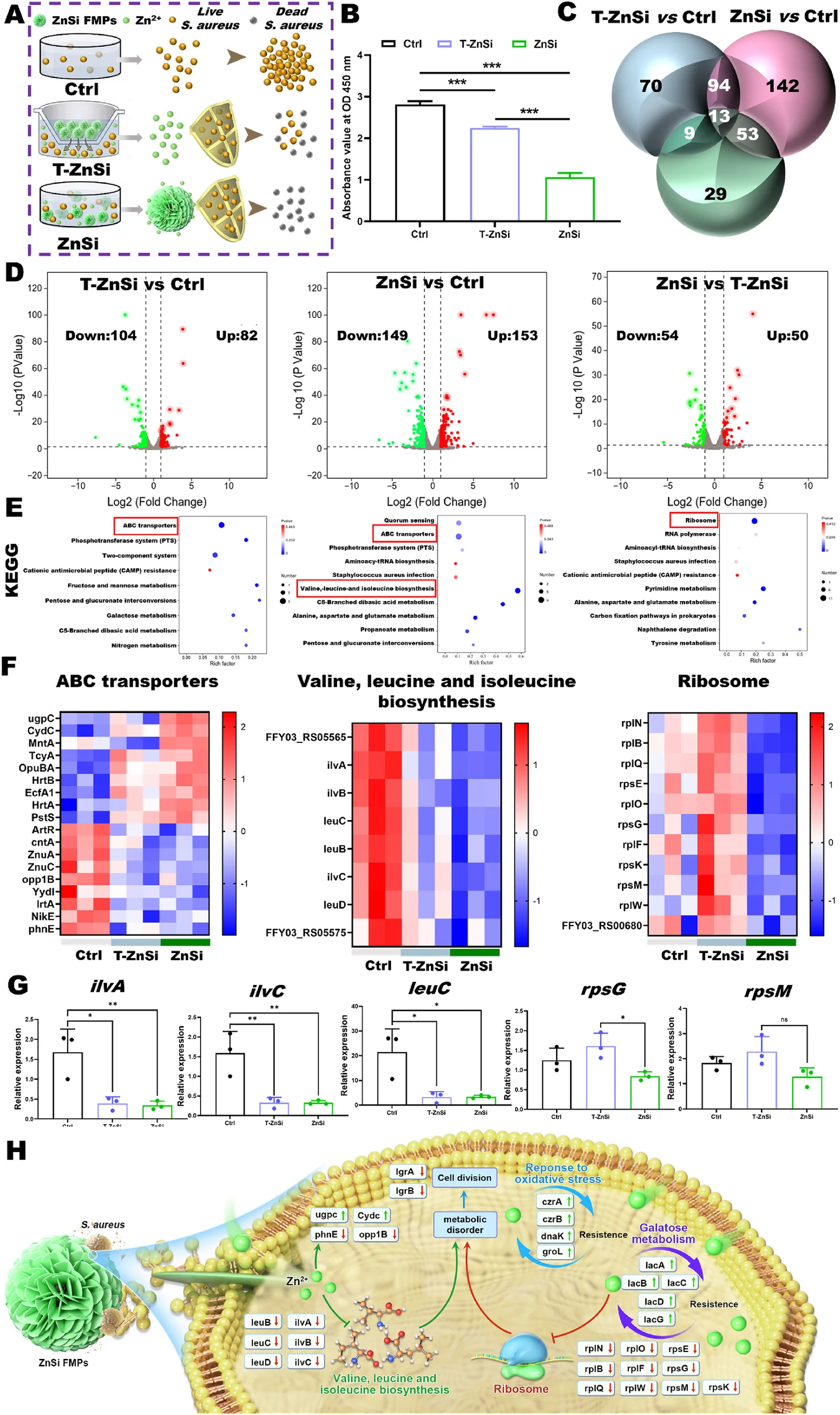
The antibacterial mechanism of ZnSi FMPs (n = 3). Control groups: Ctrl and T-ZnSi. Experimental groups: ZnSi. (A) Scheme of *S. aureus* cultured with samples. (B) Absorbance results of *S. aureus* cultured with samples. ***p < 0.001, by one-way ANOVA with Bonferroni *post hoc* test. (C) Venn diagram of DEGs, (D) Volcano diagrams of DEGs of T-ZnSi *vs* Ctrl, ZnSi *vs* Ctrl and ZnSi *vs* T-ZnSi, (E) KEGG enrichment analyses of down-regulated DEGs of T-ZnSi *vs* Ctrl, ZnSi *vs* Ctrl and ZnSi *vs* T-ZnSi. (F) Heatmaps of down-regulated DEGs involved in *ABC transporter* pathway, *Valine, leucine and isoleucine biosynthesis* pathway and *Ribosome* pathway of three groups (Ctrl, T-ZnSi and ZnSi). (G) Relative expression of *ilvA*, *ilvC*, *leuC*, *rpsG* and *rpsM* of *S. aureus* cultured with three groups (Ctrl, T-ZnSi and ZnSi) (n = 3). *p < 0.05, **p < 0.01, by one-way ANOVA with Bonferroni *post hoc* test. (H) Schematic diagram of antibacterial mechanism of ZnSi FMPs.

The edges of two-dimensional nanosheets have been reported to physically damage bacterial cells by inserting into and disrupting the cell membrane upon direct contact, resulting in membrane disruption, leakage of cytoplasmic contents, and eventual cell death [25]. SEM images of *S. aureus* as shown in Fig. 2F and G revealed that dead bacteria cells were consistently localized on the nanosheets of ZnSi FMPs, suggesting that the nanosheets may exert antibacterial effects through direct physical interaction. To elucidate the underlying antibacterial mechanism of ZnSi FMPs, the contributions of physical interaction and Zn^2+^ release were systematically investigated. *S. aureus* was cultured with a control group (PBS), a non-contact group(T-ZnSi, ZnSi FMPs evenly distributed on a 24-well polycarbonate Transwell insert) and a direct group (ZnSi, ZnSi FMPs evenly distributed on a 24-well plate) for 6 h, respectively, as illustrated in Fig. 3A. The supernatant and bacterial suspensions were then collected for the following analyses. Absorbance measurements as shown in Fig. 3B revealed that both T-ZnSi and ZnSi presented significant antibacterial activities compared to the Ctrl group, and the ZnSi group demonstrated superior efficacy, with a statistically significant reduction in bacterial viability compared to the T-ZnSi (P < 0.001). Meanwhile, Zn ion quantification of supernatant as shown in Fig. S5 revealed comparable Zn^2+^ concentrations for T-ZnSi and ZnSi, with no significant difference. This observation indicated that direct surface contact between ZnSi FMPs and bacteria might enhance the antibacterial performance of ZnSi FMPs. RNA sequencing was then performed to identify the differentially expressed genes (DEGs) among all the groups. The DEGs Venn diagram and Volcano diagrams were shown in Fig. 3C and D. For T-ZnSi vs Ctrl, 186 DEGs were found including 82 up-regulated genes and 104 down-regulated genes. For ZnSi vs Ctrl, 302 DEGs were detected including 153 up-regulated genes and 149 down-regulated genes. For ZnSi vs T-ZnSi, 104 DEGs were found including 50 up-regulated genes and 54 down-regulated genes. The heatmap of the top up- and down-regulated DEGs of three groups were shown in Fig. S6, especially, the expression of metal ion efflux-related genes (*czrA* and *czrB)* and the oxidative stress-related gene (*dnaK* and *groL)* in both T-ZnSi and ZnSi groups were significantly elevated compared to the Ctrl group, indicating a common Zn^2+^-induced stress response. Moreover, the T-ZnSi group specifically up-regulated the expression of pyrimidine synthesis genes (*carB*, *pyrE* and *pyrF*), while the ZnSi group specifically up-regulated the expression of oxidative stress-related gene (*clpB*) and down-regulated the expression of ribosomal protein and amino acid synthesis genes (*serS, rplN, rplB* and *rplQ*).

Gene Ontology (GO) enrichment analyses of DEGs as shown in Fig. S7A and Fig. S7B revealed that the down-regulated genes of T-ZnSi *vs* Ctrl were mainly enriched in membrane integrity (*membrane, integral component of membrane, intrinsic component of membrane*) and transport functions (*transport, metal ion transport, transmembrane transporter activity, ABC-type transporter activity*), the up-regulated genes of T-ZnSi *vs* Ctrl were mainly enriched in nucleotide and small molecule metabolism (*nucleobase-containing small molecule metabolic process, nucleotide metabolic process, glycerol metabolic process, pyrimidine nucleobase metabolic process*). For ZnSi *vs* Ctrl, the down-regulated genes of DEGs were mainly enriched in amino acid biosynthesis (*branched-chain amino acid biosynthetic and metabolic processes, leucine biosynthetic and metabolic processes, cellular amino acid metabolic process*), ribosomal protein synthesis (*ribosomal subunit, large ribosomal subunit*) and transporter activity (*active transmembrane transporter activity, ABC-type transporter activity, ATPase-coupled transmembrane transporter activity*), the up-regulated genes of DEGs were mainly enriched in nucleotide and small molecule metabolism (*lactose metabolic and catabolic processes, oligosaccharide catabolic process, disaccharide catabolic process*), *protein folding* and transporter activity (secondary *active transmembrane transporter activity, active transmembrane transporter activity, active ion transmembrane transporter activity*). For ZnSi *vs* T-ZnSi, the down-regulated genes of DEGs were mainly enriched in ribosome biogenesis (*ribosome, structural constituent of ribosome*), and the up-regulated genes of DEGs were mainly enriched in energy metabolism process (*lactose metabolic and catabolic processes, oligosaccharide catabolic process, disaccharide metabolic and catabolic processes*).

KEGG enrichment analyses of DEGs were then performed and showed in Fig. 3E and Fig. S7C, the down-regulated DEGs showed that T-ZnSi group significantly disrupted transport system (*ABC Transporters*), while the up-regulated DEGs mainly involved in metabolic homeostasis (*Pyrimidine metabolism*). For ZnSi vs Ctrl, the down-regulated DEGs significantly affected amino acids biosynthesis (*Valine, leucine and isoleucine biosynthesis*) and transport system (*ABC Transporters*), and the up-regulated DEGs mainly focused on metabolic homeostasis (*Galactose metabolism*) and transport system (*ABC Transporters*). For ZnSi vs T-ZnSi, the down-regulated DEGs significantly enriched in *Ribosome* pathway, while the up-regulated DEGs mainly focused on *Galactose metabolism* pathway. Fig. 3F and Fig. S8 illustrated the heatmap of DEGs involved in *ABC transporter* pathway, *Valine, leucine and isoleucine biosynthesis* pathway, *Ribosome* pathway, *Pyrimidine metabolism* pathway and *Galactose metabolism* pathway of three groups (Ctrl, T-ZnSi and ZnSi). In the *ABC transporter* pathway, both the T-ZnSi and ZnSi groups showed different expression compared to the Ctrl group, and the ZnSi group specifically up-regulated the expression of *ugpC* (involved in glycerol-3-phosphate transport), *CydC* and *MntA* (involved in metal ion transport). For the *Valine, leucine and isoleucine biosynthesis* pathway and *Ribosome* pathway, ZnSi FMPs displayed distinct down-regulation on the synthesis of *valine, leucine, isoleucine* and *ribosome* compared to the Ctrl group, indicating that ZnSi exerts a more significant influence on the synthesis and functional regulation of *S. aureus.* In the *Pyrimidine metabolism* pathway, the T-ZnSi group specifically up-regulated the expression of *carB*, *pyrE* and *pyrF*, whereas the ZnSi group specifically up-regulated the expression of *lacA*, *lacB*, *lacC*, *lacD* and *lacG* in the *Galactose metabolism* pathway.

Consistent with the transcriptional trends shown in Fig. 3F, the quantitative qRT-PCR results in Fig. 3G further verified the differential expression of representative hub genes from *Valine, leucine and isoleucine biosynthesis* pathway and *Ribosome* pathway. Compared with the Ctrl group, the key biosynthetic genes of *valine, leucine and isoleucine ilvA*, *ilvC* and *leuC* were markedly suppressed in both T-ZnSi and ZnSi groups, with the ZnSi group exhibiting the lowest relative expression levels. For ribosomal structural genes, the expression of *rpsG* and *rpsM* of T-ZnSi were up-regulated compared with the Ctrl groups, while the ZnSi group also exhibited the lowest relative expression levels among them. The results further confirmed that ZnSi FMPs strongly interfered with bacterial branched-chain amino acid biosynthesis and ribosome assembly at the transcriptional level.

In general, the results of RNA sequencing revealed that both T-ZnSi and ZnSi achieved favorable antibacterial activity through Zn^2+^-mediated stress responses to up-regulate the expression of *czrA*, *czrB*, *dnaK* and *groL*. Beyond these common stress responses, GO and KEGG analyses indicated that T-ZnSi primarily inhibited bacterial activity through disrupting *ABC transporters* and *Pyrimidine metabolism*. Notably, as depicted in the schematic in Fig. 3H, the synergy of direct physical contact and sustained Zn^2+^ release enabled ZnSi FMPs to execute a comprehensive metabolic interference. This effect was characterized by the simultaneously compromising *ABC transporters* and blocking essential biosynthetic processes including the synthesis of *valine, leucine, isoleucine* and *ribosome*, and *Galactose metabolism* pathway was also activated for resistance. These multi-pathway transcriptional interference enables ZnSi FMPs to serve as promising functional antibacterial agent for wound therapy.

### 2.4 Anti-inflammatory and angiogenesis of ZnSi FMPs *in vitro*

**Fig. 4:**
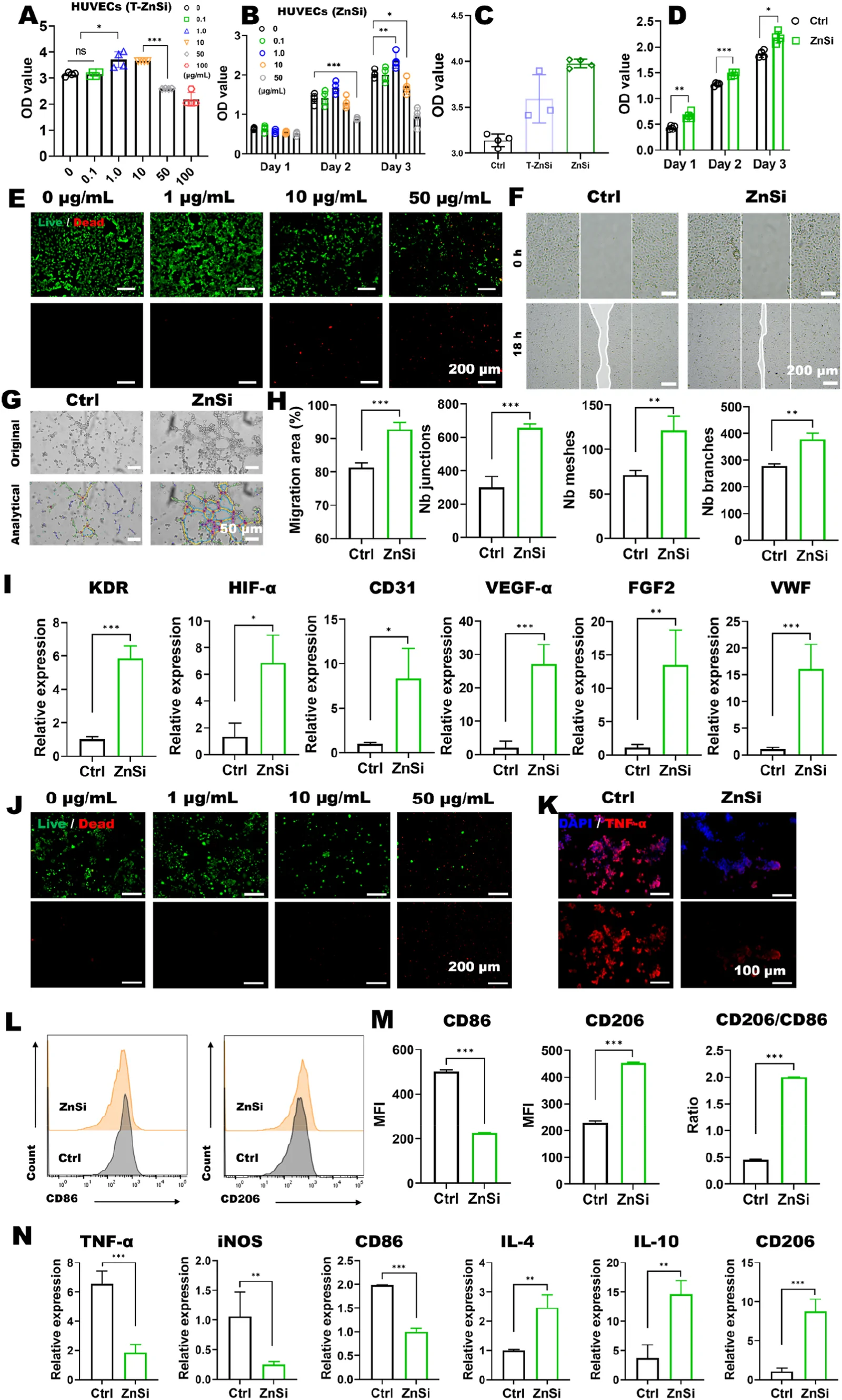
(A-I) Angiogenesis of ZnSi FMPs *in vitro*. (A) Cytotoxicity results of HUVECs cultured with different T-ZnSi content (n = 4). *p < 0.05, ***p < 0.001, by one-way ANOVA with Bonferroni *post hoc* test. (B) Cytotoxicity results of HUVECs cultured with different ZnSi content (n = 4). **p < 0.05, ***p < 0.001, by two-way ANOVA with Bonferroni *post hoc* test. (C) Cytotoxicity results of HUVECs cultured with Ctrl, T-ZnSi and ZnSi groups (n = 4). (D) Cell proliferation assays of HUVECs cultured with ZnSi (n = 4). *p < 0.05, **p < 0.01, ***p < 0.001, by two-way ANOVA with Bonferroni *post hoc* test. (E) Live/dead staining of HUVECs cultured with different ZnSi content. (F) Cell migration and (G) Tube formation images of HUVECs cultured with ZnSi FMPs. (H) Quantitative results of cell migration and tube formation of HUVECs cultured with ZnSi FMPs (n = 4). **p < 0.01, ***p < 0.001, by one-way ANOVA with Bonferroni *post hoc* test. (I) Relative expression of vascular-related genes of HUVECs cultured with ZnSi FMPs (n = 4). *p < 0.05, **p < 0.01, ***p < 0.001, by one-way ANOVA with Bonferroni *post hoc* test. (J-N) Anti-inflammatory of ZnSi FMPs *in vitro*. (J) Live/dead staining of RAW 264.7 cultured with different ZnSi content. (K) IF staining of *TNF-α*/DAPI, (L) Flow cytometry of *CD86* and *CD206*, (M) Quantitative intensity of *CD86* and *CD206* (n = 3). ***p < 0.001, by one-way ANOVA with Bonferroni *post hoc* test, and (N) Relative expression of inflammation-related genes of RAW 264.7 cultured with ZnSi FMPs (n = 3). **p < 0.01, ***p < 0.001, by one-way ANOVA with Bonferroni *post hoc* test.

To evaluate the angiogenic and anti-inflammatory potentials of ZnSi FMPs, the effects of ZnSi content and material morphology on human umbilical vein endothelial cells (HUVECs) were firstly investigated as shown in Fig. 4A, B and 4E. HUVECs cultured with varying concentrations of T-ZnSi as shown in Fig. 4A demonstrated the optimal cell activity at both 1 μg/mL and 10 μg/mL conditions, whereas direct exposure to ZnSi FMPs as shown in Fig. 4B and E revealed that 1 μg/mL ZnSi FMPs clearly promoted cell proliferation. Hence, subsequent experiments compared the viability of the Ctrl, T-ZnSi (1 μg/mL) and ZnSi (1 μg/mL) after 2 days of culture. The results in Fig. 4C showed both T-ZnSi and ZnSi enhanced cell proliferation, with ZnSi exhibiting superior stimulatory effects compared to T-ZnSi, indicating the flower-like morphology microstructure of ZnSi FMPs provides favorable topographical cues for endothelial cell growth. Therefore, 1 μg/mL ZnSi was selected for further mechanistic studies. Cell proliferation assays as shown in Fig. 4D demonstrated that ZnSi FMPs could promote cell growth during culturation. Moreover, cell migration, tube formation and q-PCR experiments were also conducted to investigated the effect of ZnSi FMPs on angiogenesis as shown in Fig. 4F–I. It could be concluded that ZnSi group exhibited a favorable effect on angiogenesis, particularly in upregulating key vascular genes *VEGF-α, FGF2* and *VWF*, whose expression levels were significantly higher than the Ctrl group.

Meanwhile, the immunomodulatory properties of ZnSi FMPs were evaluated using RAW 264.7 macrophages. Cytotoxicity test as shown in Fig. S9 and Fig. 4J confirmed that 1 μg/ml ZnSi FMPs supported optimal macrophage viability and activity. Immunofluorescence (IF) staining for the pro-inflammatory cytokine *TNF-α* as shown in Fig. 4K demonstrated that ZnSi FMPs significantly inhibited its expression. Flow cytometry analysis of macrophage polarization markers was further conducted, and the proportion of the surface markers *CD86*^+^ (M1) and *CD206*^+^ (M2) was recorded as show in Fig. 4L and M. The results indicated that ZnSi FMPs significantly decreased the proportion of *CD86*^+^ (M1-like) cells and increased the proportion of *CD206*^+^ (M2-like) cells, and the *CD206/CD86* ratio in the ZnSi group was over 3-fold higher than that of the Ctrl group, indicating a potent macrophage polarization toward the M2 phenotype. Moreover, qRT-PCR was performed to explore the expression of M1 macrophage markers, *TNF-α, iNOS*, and *CD86*, and M2 markers, *IL-4, IL-10* and *CD206*, and the results were shown in Fig. 4N. It could be concluded that ZnSi FMPs exhibited the optimal ability to inhibit the expression of *TNF-α, iNOS* and *CD86* and promote the expression of *IL-4, IL-10* and *CD206*, compared to the Ctrl group, indicating a satisfactory synergistic effect of Zn^2+^, SiO_4_^4−^ ions and flower-like morphology.

### 2.5 Therapeutic effect of ZnSi FMPs on *S. aureus*-infected chronic wounds in mice

**Fig. 5:**
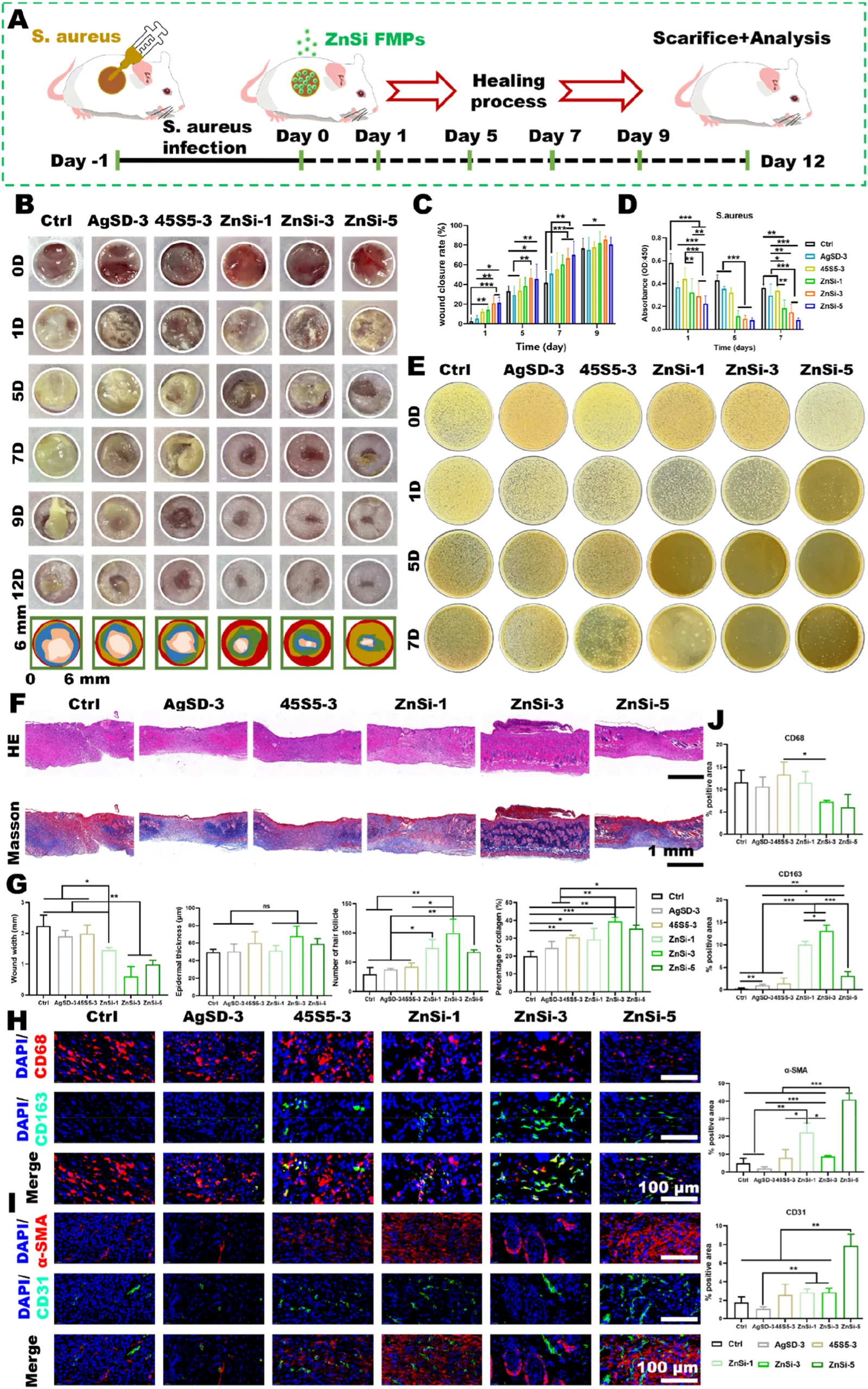
Treatment of *S. aureus*-infected wound with ZnSi FMPs in mice. Control groups: Ctrl, AgSD-3 and 45S5-3. Experimental groups: ZnSi-1, ZnSi-3 and ZnSi-5. (A) Schematic illustration of the modeling and treatment steps. (B) Optical images and wound size markers on day 1, 5, 7, 9 and 12. (C) Wound closure rate on day 1, 5, 7 and 9 (n = 6). *p < 0.05, **p < 0.01, ***p < 0.001, by two-way ANOVA with Bonferroni *post hoc* test. *S. aureus* of (D) Absorbance (n = 6). *p < 0.05, **p < 0.01, ***p < 0.001, by two-way ANOVA with Bonferroni *post hoc* test, and (E) Optical images on day 1, 5 and 7 (n = 6). (F, G) HE, Masson staining and related statistical quantification of wounds on day 12 (n = 3). *p < 0.05, **p < 0.01, ***p < 0.001, by one-way ANOVA with Bonferroni *post hoc* test. (H-J) IF staining of (H) *CD68/CD163* and (I) *α-SMA/CD31* and related statistical quantification of wounds on day 12 (n = 3). *p < 0.05, **p < 0.01, ***p < 0.001, by one-way ANOVA with Bonferroni *post hoc* test.

A *S. aureus*-infected mice wound model was established to assess the therapeutic effect of ZnSi FMPs as shown in Fig. 5A. Wound closure was monitored over 12 days, with representative macroscopic images shown in Fig. 5B and quantitative analyses in Fig. 5C. On day 0, all the particles were evenly distributed in each infected wound. On 1st day, all ZnSi groups started to show better would closure rates than the other groups. This advantage became more pronounced by day 5, particularly in the ZnSi-3 and ZnSi-5 groups. The superior healing performance of ZnSi FMPs persisted throughout the 12-day observation period, with the ZnSi-3 demonstrating the most rapid wound closure among all groups, consistent with the statistical data in Fig. 5C. Moreover, wound exudates were collected on days 1, 5, and 7 for bacterial counting as shown in Fig. 5D and agar plate culture in Fig. 5E. On day 0, no significant difference was found in each group. However, by day 1, all ZnSi FMPs groups exhibited better antibacterial property than the other three groups, with a clear dose-dependent effect. This suppression of bacterial viability was sustained over 7 days, confirming the potent and durable antibacterial action of ZnSi FMPs.

Histological analyses were performed to evaluate the biosafety of ZnSi FMPs and the functional repair of regenerated skin tissue. Firstly, HE staining of heart, liver, spleen, lung and kidney tissues as shown in Fig. S10 indicated that all groups had good biocompatibility, no obvious histopathological changes including necrosis, tissue disorganization and inflammation observed in any of the major organs. HE and Masson staining of wound tissues were also conducted as shown in Fig. 5F, the length of wound tissue, wound epithelial thickness, the number of newly hair follicles, and the percentage of newly collagen were counted as shown in Fig. 5G. According to the statistical results, the wound length of all ZnSi FMPs groups were significantly lower than the other three control groups with statistical difference, and ZnSi-3 group showed the smallest residual wound length, which were consistent with the wound closure rate results as shown in Fig. 5C. For the wound epithelial thickness, ZnSi-3 also exhibited the thickest neo-epidermis, indicating robust re-epithelialization. Furthermore, hair follicle neogenesis was markedly enhanced in all ZnSi groups, with ZnSi-3 group had the highest density. The newly collagen could be stained blue in Masson staining as shown in Fig. 5F, the AgSD-3 and 45S5-3 showed a promotion effect on collagen regeneration, and all ZnSi groups showed significantly promotion effect with statistical differences, while ZnSi-3 group exhibited the optimal effect on collagen amount and the degree of orderly arrangement, indicating a favorable effect on wound healing.

Moreover, Immunohistochemical and IF staining were performed to evaluate the effect of ZnSi on anti-inflammatory (*CD68/CD163*) and vascular regeneration (*α-SMA/CD31*) as shown in Fig. 5H and I, and quantitative analyses were shown in Fig. 5J. In immunofluorescence staining of inflammatory response, red fluorescence for *CD68* served as a pan-macrophage marker to identify all macrophages, whereas green fluorescence for *CD163* was employed as a specific marker for M2-polarized macrophages. Notably, the positive area of *CD68* in the Ctrl, AgSD-3 and 45S5-3 groups were higher than all ZnSi groups, whereas the positive area of *CD163* in the Ctrl, AgSD-3 and 45S5-3 groups were much lower than all ZnSi groups, especially in the ZnSi-3 group. These findings confirmed that ZnSi FMPs effectively modulated the inflammatory microenvironment toward a healing-promoting state. Moreover, the expression results of *α-SMA* and *CD31* showed that the ZnSi groups could significantly promoted microvessel formation, while the ZnSi-1 and ZnSi-5 groups promoted the expression of *α-SMA*, and the ZnSi-5 group promoted the expression of *CD31*.

### 2.6 Investigation into the therapeutic mechanism of ZnSi FMPs in *S. aureus*-infected wounds

**Fig. 6:**
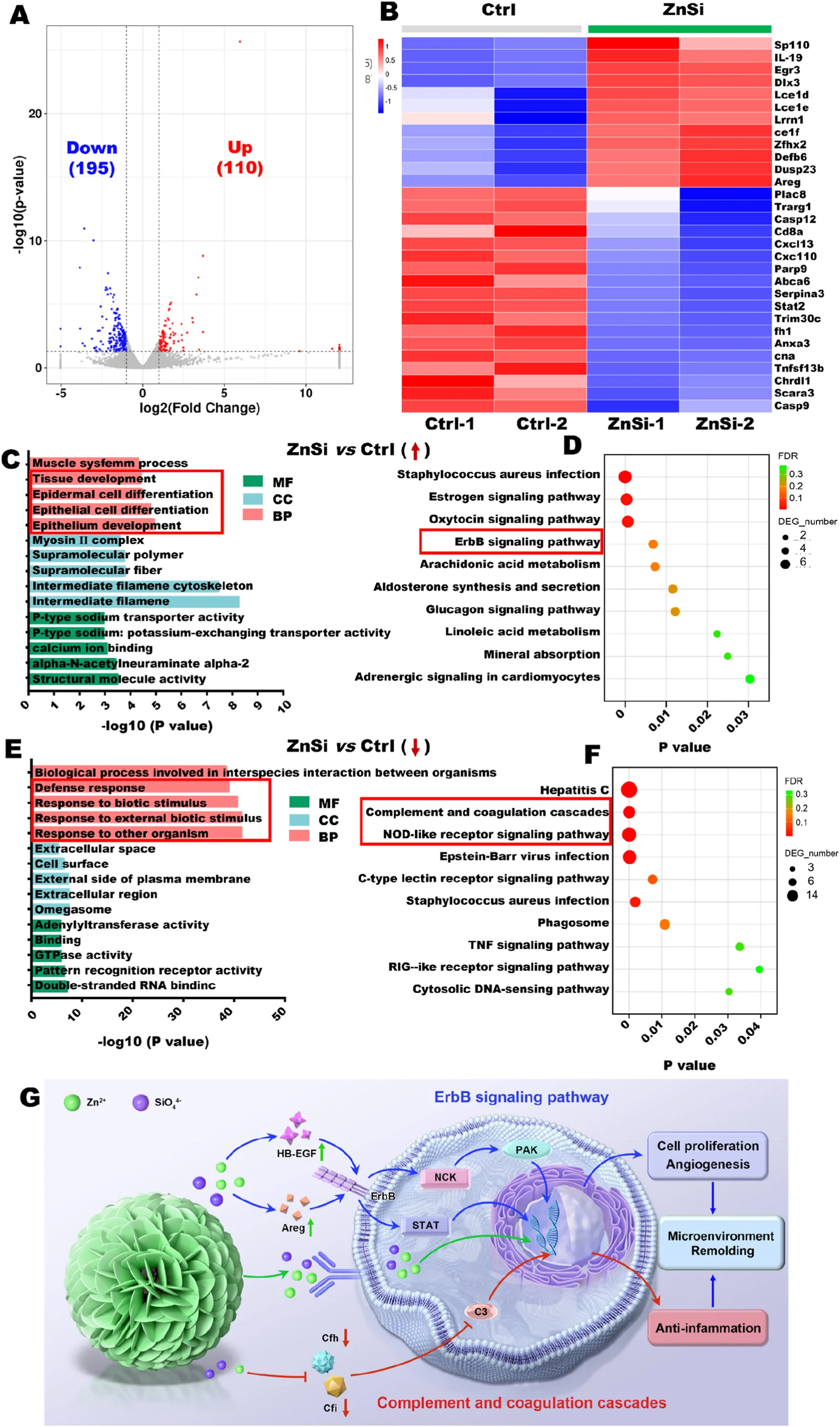
RNA sequencing of ZnSi FMPs on *S. aureus*-infected chronic wounds (n = 2). (A) Volcano plot. (B) Heatmap. (C) GO enrichment analysis and (D) KEGG enrichment analysis of up-regulated DEGs. (E) GO enrichment analysis and (F) KEGG enrichment analysis of down-regulated DEGs. (G) Scheme of therapeutic mechanism of ZnSi FMPs on wound healing.

To investigate the mechanism of ZnSi FMPs on *S. aureus*-infected chronic wounds healing, RNA sequencing was performed of the Ctrl and ZnSi groups at day 7 post-injury in mice. A total of 305 DEGs were identified, including 195 down-regulated and 110 up-regulated genes in the ZnSi group compared to the Ctrl group as shown in Fig. 6A. Moreover, the heatmap of 30 DEGs about inflammatory response and wound healing among top 60 DEGs was shown in Fig. 6B. For the term of bacterial infection, the markers of bacterial infection, *Serpina3f* and *Trim30c*, of the ZnSi group were significantly lower than that of the Ctrl group, and the mRNA expression of antibacterial, *Defb6* and *Sp110* of the ZnSi group were much higher than that of the Ctrl group. For the term of inflammatory response, the marker of inflammation, *Anxa3*, of the ZnSi group were significantly lower than that of the Ctrl group, the pro-inflammatory genes, including *Parp9, Stat2, Tnfsf13b, Ifih1, Cxcl13,* and *Cxcl10* of ZnSi group were significantly lower than that of the Ctrl group, and the anti-inflammatory genes, *Areg*, *Dusp23,* and *IL-19* of the ZnSi group were much higher than that of the Ctrl group. Furthermore, the expression of genes related to epithelial repair and skin regeneration, *Areg*, *Egr3, Lce1d, Lce1f, Lce1e, Dlx3* and *Lrrn1* of the ZnSi group were much higher than that of the Ctrl group.

GO analyses of up-regulated DEGs as shown in Fig. 6C mainly related to tissue regeneration (*epidermis development, epidermal cell differentiation, epithelial cell differentiation* and *tissue development*), while down-regulated DEGs as shown in Fig. 6E mainly related to immune response (*defense response, response to external biotic stimulus*). KEGG analyses as shown in Fig. 6D and F showed the same trend with GO analyses, the up-regulated DEGs mainly related to tissue regeneration, *ErbB signaling pathway*. Moreover, the down-regulated DEGs mainly related to immune response (*Complement and coagulation cascades* and *NOD-like receptor signaling pathway*). The results indicated that ZnSi FMPs could decrease the level of inflammatory response via inhibiting the expression of pro-inflammatory genes, and promote wound healing by enhancing the expression of related genes. Hence, the scheme of promotion mechanism of ZnSi FMPs on wound healing could be concluded as shown in Fig. 6G, with the release of Zn^2+^ and SiO_4_^4−^, up-regulated *Areg* and *HB-EGF* combined receptors on the surface of cell membranes to activate *ErbB signaling pathway* to promote wound healing, whereas *Cfh*, *Cfi* and *C3* were down-regulated and activated *Complement and coagulation cascades* to suppress inflammation response.

### 2.7 Therapeutic effect of ZnSi FMPs on diabetic chronic wounds in mice

**Fig. 7:**
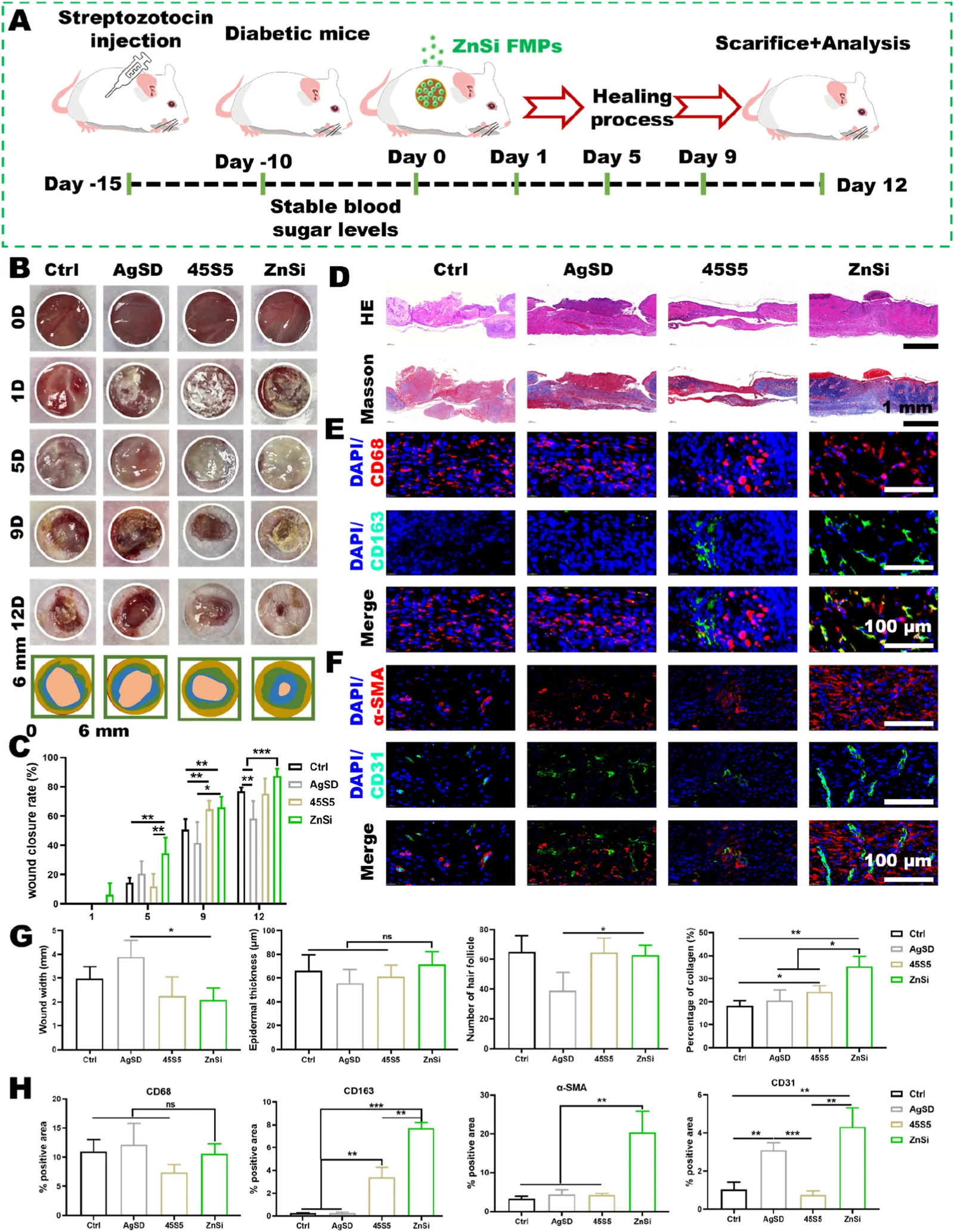
Treatment of diabetic chronic wound with ZnSi FMPs in mice. Control groups: Ctrl, AgSD and 45S5. Experimental groups: ZnSi. (A) Schematic illustration of the modeling and treatment steps. (B) Optical images, wound size markers and (C) Wound closure rate on day 1, 5, 9 and 12 (n = 6). *p < 0.05, **p < 0.01, ***p < 0.001, by two-way ANOVA with Bonferroni *post hoc* test. (D, G) HE, Masson staining and related statistical quantification of wounds on day 12 (n = 3). *p < 0.05, **p < 0.01, by one-way ANOVA with Bonferroni *post hoc* test. (E, F and H) IF staining of *CD68/CD163* and *α-SMA/CD31* and related statistical quantification of wounds on day 12 (n = 3). *p < 0.05, **p < 0.01, ***p < 0.001, by one-way ANOVA with Bonferroni *post hoc* test.

To further evaluate the therapeutic potential of ZnSi FMPs on impaired wound healing, a diabetic wound model was established as shown in Fig. 7A, the macroscopic changes of wounds were illustrated in Fig. 7B, with statistical analyses illustrated in Fig. 7C. The wounds showed no significant differences on day 0 and day 1. On 5th day, purulent exudate could be observed on all wound surface, indicating a pronounced inflammatory response. On 9th day, purulent exudate could still be observed on the surface of the Ctrl and the AgSD groups, while the 45S5 and the ZnSi groups showed favorable effects on wound healing with significant statistical difference. On 12th day, re-ulceration of the wound tissue was observed in the Ctrl, AgSD and 45S5 groups, whereas the ZnSi group exhibited favorable healing effect, indicating ZnSi FMPs exerted a beneficial effect on chronic wound healing.

The representative H&E, Masson staining and the statistical analyses including the length of wound tissue, epithelial thickness, the number of newly hair follicles and the percentage of newly collagen as shown in Fig. 7D and G illustrated the difficulty of healing diabetic chronic wounds, all the wound tissue of the Ctrl, AgSD and 45S5 groups exhibited a discontinuous structure, with limited regeneration of collagen fibers. In contrast, the healing wound tissue of the ZnSi group demonstrated a dense structure, with amount of continuous arranged collagen fibers.

The immune response and vascular regeneration of wound tissues were also explored as shown in Fig. 7E, F and 7H. Severe immune response could be detected in the Ctrl and the AgSD group, with high positive area of *CD68* and low positive area of *CD163*. The 45S5 group also exhibited certain anti-inflammatory effects, while the ZnSi FMPs exhibited excellent anti-inflammatory effects with high positive area of *CD163*, indicating the superior ability of ZnSi on anti-inflammation. The vascular regeneration demonstrated that ZnSi FMPs also significantly promoted the expression of vascular related genes, especially the expression of *α-SMA*, which was far higher than the other three groups.

### 2.8 Therapeutic effect of ZnSi FMPs on *S. aureus*-infected chronic wounds in minipigs

**Fig. 8:**
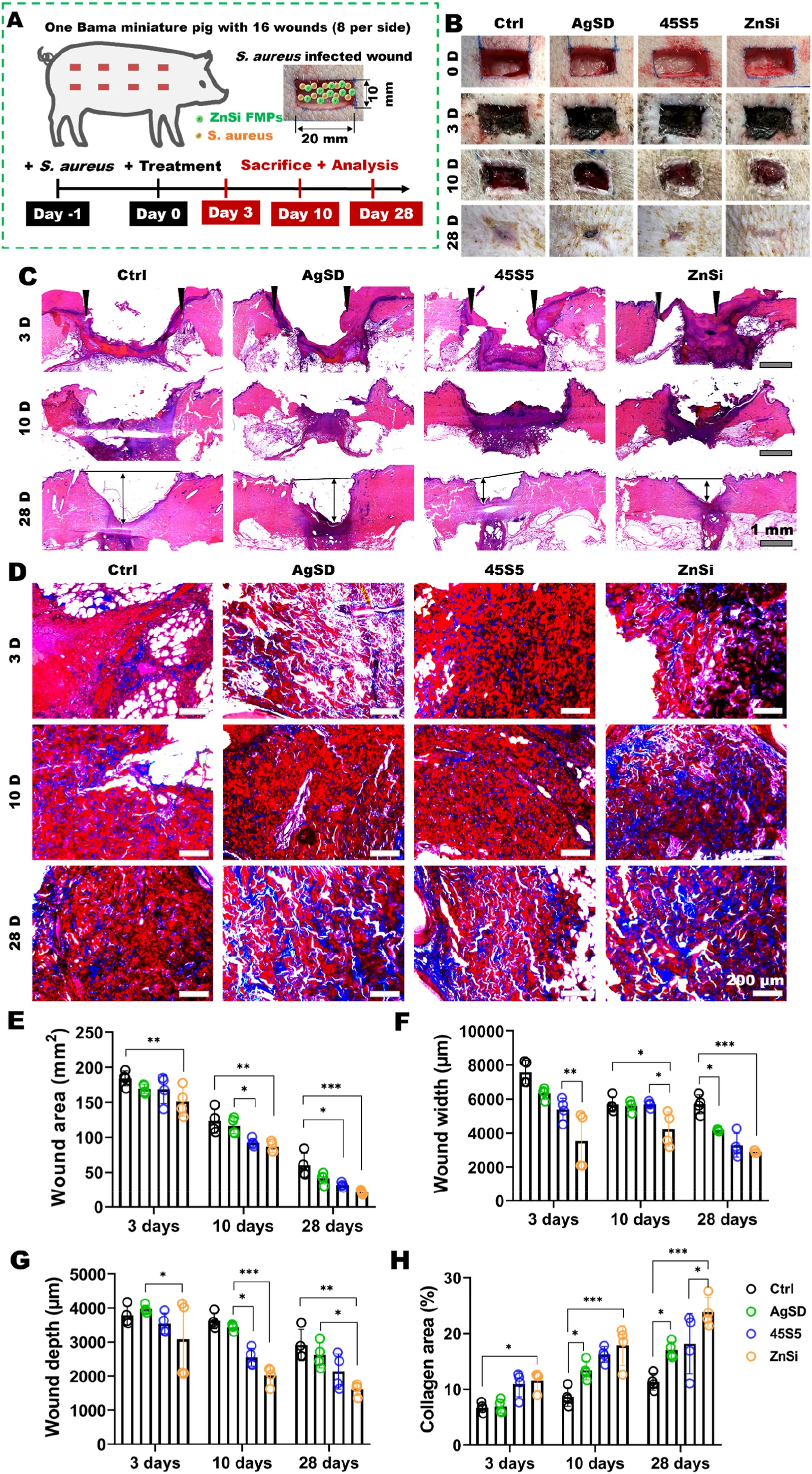
Treatment of *S. aureus*-infected wound with ZnSi FMPs in minipigs. Control groups: Ctrl, AgSD and 45S5. Experimental groups: ZnSi. (A) Schematic illustration of the modeling and treatment steps. (B) Optical images and (E) Wound area on day 3, 10 and 28 (n = 4). *p < 0.05, **p < 0.01, ***p < 0.001, by two-way ANOVA with Bonferroni *post hoc* test. (C) HE, (D) Masson staining and (F-H) Related statistical quantification of wounds on day 3, 10 and 28 (n = 4). *p < 0.05, **p < 0.01, ***p < 0.001, by two-way ANOVA with Bonferroni *post hoc* test.

A *S. aureus*-infected minipig wound model was established as shown in Fig. 8A to evaluate the preclinical therapeutic effect of ZnSi FMPs, considering the similar skin structure between pig and human. Wound closure was monitored over 28 days, the macroscopic images and statistical analyses of wound area were illustrated in Fig. 8B and E, on 3rd day, the ZnSi group had showed the advantage on wound healing, with the smallest residual wound area among all groups, and the trend maintained throughout the 28-day observation period.

Typical HE staining, Masson staining and related statistical analyses including wound width, depth and area were shown in Fig. 8C, D and 8F-8H. In the early stage of wound healing, ZnSi FMPs exhibited a favorable healing effect on wound width, which was significantly lower than the Ctrl, AgSD and 45S5 groups. On 10th day and 28th day, the wound depth of the ZnSi group also presented the same trend as wound width, consistent with progressive granulation tissue formation and dermal reconstruction. For the collagen regeneration, on 3rd day and 10th day, both the 45S5 and ZnSi groups exhibited comparable effects on promoting collagen regeneration, while amount of tightly and orderly arranged newly collagen were formed in ZnSi group, further validated the superiority of ZnSi on wound healing.

**Fig. 9:**
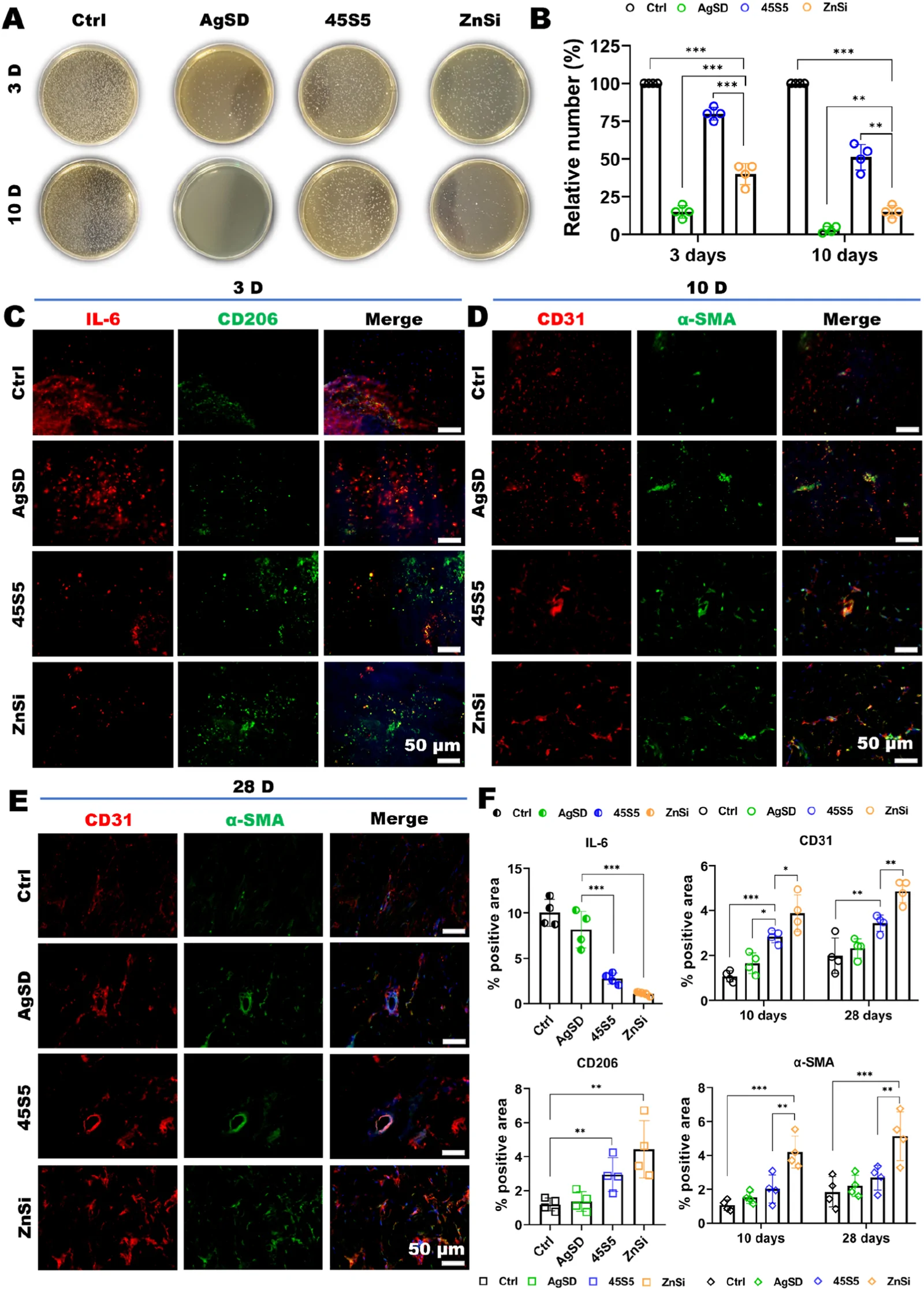
Treatment of *S. aureus*-infected wound with ZnSi FMPs in minipigs. Control groups: Ctrl, AgSD and 45S5. Experimental groups: ZnSi. *S. aureus* of (A) Optical images and (B) Relative number on day 3 and 10 (n = 4). **p < 0.01, ***p < 0.001, by two-way ANOVA with Bonferroni *post hoc* test. (C-F) IF staining of *IL-6/CD206* on day 3, *CD31/α-SMA* on day 10 and 28 and related statistical quantification of wounds (n = 4). *p < 0.05, **p < 0.01, ***p < 0.001, by two-way ANOVA with Bonferroni *post hoc* test.

For the antibacterial capacity as shown in Fig. 9A and B, both the AgSD and ZnSi groups exhibited good antibacterial property on 3rd day and 10th day. Meanwhile, IF staining of inflammatory factors (*IL-6*, *CD206*) at initial stage and pro-angiogenic factors (*CD31*, *α-SMA*) at late stage as shown in Fig. 9C–F. The results demonstrated that ZnSi FMPs could significantly inhibit the expression of pro-inflammatory factors IL-6 and promote the expression of anti-inflammatory factors *CD206* compared to the other three groups, indicating the favorable effect of ZnSi FMPs on anti-inflammation. In parallel, the expression of *CD31* and *α-SMA* also exhibited the advantages of ZnSi FMPs on vascularization, especially the expression of *α-SMA*, the positive area of *α-SMA* of ZnSi on 10th day and 28th day was more than twice as much as the other three groups.

## 3 Discussion

Infection susceptibility and impaired healing are two major challenges in the repair of chronic wounds [26]. However, current therapeutic strategies typically fail to simultaneously address both bacterial infection and tissue regeneration, leading to poor clinical outcomes and prolonged healing times. The use of Zn^2+^ and silicate ions for antibacterial, pro-angiogenic, anti-inflammatory, and wound healing purposes has been widely reported, and zinc silicate also has been applied in tissue repair. Nevertheless, apart from ion release behavior, material microstructure also exerts a vital regulatory impact on biological performance. In this work, we have developed hierarchical zinc silicate flower-like microspheres (ZnSi FMPs) via a controlled hydrothermal method. The present study has shown that the synergistic effect of nanosheet structure and Zn^2+^ release endow ZnSi FMPs with favorable antibacterial property, and the release of Zn^2+^ and SiO_4_^4−^ could regulate inflammation and promote vascularization during the healing process of chronic wounds.

We prepared a novel well-assembled and well-distributed ZnSi FMPs using hydrothermal method, and investigated the sources of raw materials, the content of CTAB, the reaction time and temperature to interpret the formation mechanism. As reported in previous studies, surfactants such as CTAB can significantly influence the morphology of inorganic materials by modulating the interfacial energy and electrostatic interactions during the nucleation and growth stages [27]. During the process, the concentration of CTAB was found to be a critical parameter for controlling the final particle size and morphology. At low CTAB concentrations, insufficient micelle formation resulted in a small amount of crystallization and loosely packed. Conversely, excessive CTAB led to overly stabilized micelles, which hindered the further growth and assembly of ZnSi nanosheets. Through systematic optimization, we found that a CTAB concentration of 0.3 g yielded microspheres with the most uniform size distribution and well-defined hierarchical structure, with the average size and the specific surface area were ∼3.8 μm and 41.0 m^2^/g, respectively. These structural features are beneficial for enhancing functional performance such as drug delivery and antibacterial activity, which are highly desirable for healing wound applications [28]. Moreover, the reaction time and temperature were also key factors of the formation and structural evolution of ZnSi FMPs. Prolonging the reaction time from 6 h to 24 h, along with increasing the reaction temperature from 150°C to 200°C, promoted the gradual crystallization and self-assembly of ZnSi FMPs, which could provide sufficient crystallization time and thermal energy [29]. The optimal reaction time and temperature were proven to be 24 h and 200°C, respectively. These findings have highlighted the importance of balancing kinetic and thermodynamic factors in the hydrothermal synthesis of ZnSi FMPs.

Hence, the formation mechanism of ZnSi FMPs can be summarized as shown in Fig. 1A: CTAB, as the surfactant and soft template, was firstly dissolved in the aqueous phase and formed micelles, which provided uniformly distributed nucleation sites for the initial growth of ZnSi FMPs. The positively charged hydrophilic end of the CTAB molecule electrostatically attracted the negatively charged silanol groups (Si-OH), which in turn facilitated the adsorption of positively charged Zn^2+^ ions, forming a Zn^2+^–SiO_4_^4-^ coordination complex at the interface. Importantly, under acid conditions, the ZnSi molecule is more likely to form two-dimensional nanosheets, which has a high specific surface area and low surface energy [30]. These nanosheets served as building blocks for further hierarchical assembly. As the hydrothermal reaction proceeded, the ZnSi nanosheets gradually arranged radially around the CTAB micelles, driven by electrostatic interactions and interfacial energy minimization, then led to the formation of ZnSi FMPs.

In addition, our study explored the application of ZnSi FMPs on chronic wound healing, with a particular focus on antibacterial activity, anti-inflammatory effects, and pro-healing properties. *In vitro* degradation behavior and *in vivo* HE staining of organs have proven the biosafety of ZnSi FMPs. Notably, at day 21, the cumulative released concentration of Zn ions reached 0.85 mg/L, according to previous biosafety investigations on zinc-based wound biomaterials, the recognized cytotoxic threshold of free Zn^2+^ for mammalian cells is approximately 5.2 mg/L [31], which is far higher than the ion concentration detected in this work. Moreover, a zinc silicate composite hydrogel study reported the cumulative concentration of released Zn^2+^ up to 15 mg/L within 5 days, and 14-day subcutaneous implantation assays confirmed no pathological injury to major visceral organs [32]. The mild release mode of ZnSi FMPs greatly proves the biosafety of ZnSi FMPs. The antibacterial activity of ZnSi FMPs was evaluated against Gram-positive (*Staphylococcus aureus*) bacteria and Gram-negative (*Escherichia coli*), and commercial AgSD and 45S5 powders were used as control groups. The results demonstrated that ZnSi FMPs exhibited excellent pH-independent antibacterial performance with significantly reduced bacterial viability at pH = 6.0, 7.4 and 8.5. Moreover, the antibacterial duration of ZnSi FMPs was found to be longer than that of 45S5, indicating a more sustained release profile of Zn^2+^ ion and enhanced long-term inhibition of bacterial growth.

This enhanced antibacterial performance can be attributed to the synergistic effects of sustained ion release of Zn^2+^ and the unique nanosheet topography of ZnSi FMPs. Apart from the same concentrations of T-ZnSi and ZnSi groups after treatment, RNA-sequencing results further supported this mechanism by revealing significant transcriptomic alterations in *S. aureus* upon exposure to ZnSi FMPs. Specifically, the expression of metal ion efflux-related genes *czrA* and *czrB*, as well as the oxidative stress-related genes, *dnaK* and *groL*, were markedly up-regulated in both T-ZnSi and ZnSi groups compared to the Ctrl group, indicating that Zn^2+^ induced a strong intracellular stress response in bacteria. In particular, the ZnSi group significantly down-regulated genes associated with ribosomal subunits (*rplN*, *rplB*, *rplQ*) and amino acid synthesis (*serS*)*,* suggesting ZnSi FMPs imposed both oxidative stress and severe metabolic dysregulation. GO and KEGG enrichment analyses provided deeper insights into this metabolic interference. In the T-ZnSi group, down-regulated genes were mainly enriched in *ABC transporters*—ATP-dependent transmembrane protein complexes that function as core mediators of bacterial membrane transport and nutrient acquisition [33]. The down-regulation of these transporters suggests that Zn^2+^ alone disrupts critical membrane transport, impairing bacterial nutrient acquisition and toxins clearance to perturb cellular homeostasis. In contrast, the ZnSi group, which combined nanosheet structures with Zn^2+^ release, induced broader and more severe disruptions. The down-regulated genes were enriched in *Ribosome* and *Valine*, *leucine*, and *isoleucine biosynthesis* pathways, and the qPCR results further confirmed that ZnSi FMPs strongly interfered with bacterial branched-chain amino acid biosynthesis and ribosome assembly at the transcriptional level. The ribosome encompasses genes encoding ribosomal subunits and protein synthesis, which is essential for bacterial viability [34]. Meanwhile, *valine, leucine,* and *isoleucine* are branched-chain amino acids (BCAAs)—essential building blocks for protein synthesis and bacterial growth. The concurrent down-regulation of these two pathways directly blocks protein synthesis and bacterial proliferation, effectively paralyzing bacterial viability. In addition, the up-regulated genes were mainly associated with *Galactose metabolism* pathway, a key carbon metabolism route that enables bacteria to decompose galactose into pyruvate and generate ATP for energy supply [35]. This up-regulation likely reflects a compensatory response by bacteria to mitigate energy deficiency caused by other metabolic disruptions.

These findings are consistent with previous studies on Zn^2+^- mediated antibacterial mechanisms. Fan et al. have reported that Zn^2+^ can disrupt bacterial homeostasis by inducing the upregulation of genes related to ribosomal subunit, ribosome biosynthesis, chaperone and adhesins, and the genes associated with protein folding were also up-regulated [36]. Transcriptomic analysis of *Helicobacter pylori* treated with zinc acetate have found that Zn^2+^ can inhibit *Helicobacter pylori* growth through interfering with the expression of genes related to protein synthesis [37]. Liu et al. have used transcriptome and metabonomic analyses to reveal the antibacterial mechanism of ZIF-8 nanoparticles on *Staphylococcus aureus* (MRSA), and the results have shown that ZIF-8 nanoparticles can inhibit the arginine biosynthesis pathway and induce the production of reactive oxygen species (ROS), which subsequently disrupt the tricarboxylic acid (TCA) cycle and compromise cell membrane integrity, ultimately leading to bacterial death [38]. Jia et al. have also found that Zn-0.8Li-0.5Ag alloy can inhibit the activity of MRSA via cellular metabolism disturbance and induction of ROS production [39]. Moreover, Han et al. have proven that Ag particles with different morphology exhibit distinct antibacterial effect, and transcriptome analysis further demonstrated Ag nanowires can regulate the genes related to the structure and energy metabolism of the membrane to exert antifungal effects [40]. In our study, ZnSi FMPs elicited a similar transcriptional response in *S. aureus*, and the mechanism can be summarized as shown in Fig. 3H: With the synergistic effect of unique hierarchical nanosheet structure and sustained release of Zn^2+^, *ABC transporters* are first disrupted and the expression of *ugpC*, *Cydc*, *opp1B*, *phnE*, etc. are obviously affected. Furthermore, the essential biosynthetic pathways of *Valine, leucine and isoleucine* biosynthesis pathway and *Ribosome* pathway are significantly suppressed, ultimately leading to the metabolic interference and cell division. For the resistance, the expression of metal ion efflux-related genes *czrA* and *czrB*, as well as the protein folding-related genes, *dnaK* and *groL* are significantly up-regulated, and *Galactose metabolism* pathway was also activated. Eventually, under the combined disruption of membrane integrity, protein synthesis and energy metabolism, *S. aureus* is driven toward death, and *in vivo* RNA-sequencing experiments reflected the markers of bacterial infection, *Serpina3f* and *Trim30c*, were down-regulated, and the markers of antibacterial, *Defb6* and *Sp110* were obviously up-regulated.

Regarding anti-inflammation and vascularization, ZnSi FMPs demonstrated excellent immunomodulatory and angiogenic activity *in vitro* and *in vivo*. Zn^2+^ and Silica have been shown to reduce the expression of pro-inflammatory cytokines such as *TNF-α*, *IL-1β* and *IL-6* through the inhibition of NF-κB signaling pathway [41]. Our findings, including the down-regulation of M1 markers, *TNF-α*, *iNOS* and *CD86*, and the significant up-regulation of M2 markers, *IL-4*, *IL-10*. *CD206* and *CD163*, strongly support the role of ZnSi FMPs in orchestrating the transition of the inflammatory microenvironment toward a repair-permissive phase. Meanwhile, the release of Zn^2+^ and silicate also has been proven to promote angiogenesis via the expression of VEGF and *CD31*, and *HIF-1α/VEGF* and PI3K/AKT signaling pathways might be activated during the process [42]. In addition, zinc silicate has also been applied in bone repair and wound healing, and the addition of zinc silicate has been found to promote tissue regeneration with enhanced osteogenesis and angiogenesis [23].

Our results extend these reports by showing that ZnSi FMPs significantly enhanced the proliferation, migration, and tube formation of HUVECs, accompanied by the obviously up-regulation of *VEGF-α*, *FGF2* and *VWF in vitro*. *In vivo*, the expression of *CD31* and *α-SMA* was significantly promoted across all animal models, with the ZnSi group achieving superior wound width, epidermal thickness, number of hair follicle and collagen deposition compared to Ctrl, AgSD and 45S5. Tissue RNA-sequencing of *S. aureus*-infected chronic wound further revealed the molecular landscape, showing the down-regulation of pro-inflammatory genes, *Parp9, Stat2, Tnfsf13b, Ifih1, Cxcl13,* and *Cxcl10*, alongside the up-regulation of anti-inflammatory genes, *Areg*, *Dusp23* and *IL-19*, and epithelial repair genes, *Areg*, *Egr3, Lce1d, Lce1f, Lce1e, Dlx3* and *Lrrn1*. Besides, *Casp12* and *Casp9,* genes associated with endoplasmic reticulum (ER) stress-induced apoptosis and intrinsic mitochondrial apoptosis pathways [43], respectively, were markedly down-regulated in the ZnSi group. KEGG pathway enrichment analysis revealed that these DEGs were significantly enriched in the *Complement and coagulation cascades pathway*, which are critically involved in inflammasome activation, pro-inflammatory cytokine production, and stress-induced cell death during chronic inflammation [44], suggesting a favorable effect of ZnSi FMPs on suppression of inflammation and apoptosis. In parallel, *Areg* and *HB-EGF,* the epidermal growth factor receptor (EGFR) ligand that play a central role in re-epithelialization, fibroblast activation and immune modulation [45], were obviously up-regulated in the ZnSi group compared to the Ctrl group. The up-regulation of *Areg* and *HB-EGF*, ligand of EGFR, is expected to activate the *ErbB signaling pathway*―a key regulator of epithelial cell proliferation, migration and tissue remodeling during wound repair [46], revealing the role of ZnSi FMPs in harnessing endogenous regenerative mechanisms to drive efficient healing in infected chronic wounds. To sum up, transcriptomic analysis indicated that ZnSi FMPs promoted wound healing by simultaneously suppressing *Complement and coagulation cascades pathway*-driven inflammation, while enhancing *Areg/HB-EGF*-mediated *ErbB signaling pathway*, thereby regulating the chronic wound microenvironment toward a regenerative state.

Despite the favorable wound healing capacity of ZnSi FMPs, this work has some limitations. It should be acknowledged that powder formulations are not the optimal therapeutic forms for clinical chronic wound management [47], and AgSD and 45S5 bioglass are typically incorporated as functional additives into finished dressings or ointments instead of being used as bare powders. Recent advances in biomaterial research have developed clinically friendly wound treatment platforms to overcome the drawbacks of simple powder materials [48]. The goal of this study was to investigate the intrinsic wound healing efficacy and underlying biological mechanisms of ZnSi FMPs, thereby laying a fundamental theoretical basis for subsequent clinical translation. Future studies can utilize the abundant porous structure of ZnSi FMPs to load bioactive functional drugs, and further combine the microspheres with biocompatible organic matrices such as hydrogels, microneedles and absorbable wound dressings to develop clinically applicable composite wound therapeutics.

## 4 Conclusion

In this work, a hierarchical bioactive Zn_2_SiO_4_ flower-like microsphere has been developed to effectively accelerates chronic wound healing through an integrated therapeutic strategy. The well-assembled ZnSi FMPs, featuring a uniform size distribution and hierarchical nanosheet architecture, were synthesized via a controlled hydrothermal method. ZnSi FMPs exhibited outstanding antibacterial, anti-inflammatory and pro-angiogenic activities through the synergistic effect of the unique hierarchical architecture and the sustainable release of Zn^2+^ and SiO_4_^4−^. Notably, ZnSi FMPs presented a superior therapeutic effect on chronic wounds including *S. aureus*-infected wounds and diabetic wounds in mice and minipigs to the commercial AgSD and 45S5. Mechanistically, transcriptomic analysis revealed that ZnSi FMPs execute potent bacterial metabolic interference through the disruption of *ABC transporters*, and the obstruction of essential *valine, leucine, isoleucine* and *ribosome* biosynthesis in *S. aureus*. Meanwhile, ZnSi FMPs facilitated microenvironment remodeling by suppressing *Complement and coagulation cascades pathway*-driven inflammation while activating the *Areg/HB-EGF*-mediated *ErbB signaling pathway* to facilitate tissue regeneration. In conclusion, the bioactive Zn_2_SiO_4_ flower-like microsphere represent a highly promising therapeutic platform for chronic wound healing, with significant potential for clinical translation.

## CRediT authorship contribution statement

**Fanyan Deng:** Writing – original draft, Validation, Methodology, Investigation, Funding acquisition, Formal analysis, Data curation, Conceptualization. **Zizhuo Liu:** Methodology, Investigation, Data curation. **Yiran Shao:** Resources, Methodology. **Xiude Chen:** Investigation. **Kexin Zhang:** Investigation. **Yanping Feng:** Investigation. **Jinyu Zeng:** Resources. **Dong Han:** Writing – review & editing, Resources, Project administration. **Shunxiang Xu:** Writing – review & editing, Validation, Methodology, Investigation, Funding acquisition, Formal analysis, Data curation. **Congqin Ning:** Writing – review & editing, Supervision, Resources, Project administration, Conceptualization.

## Ethics approval and consent to participate

The animal researches involving mice were approved by the Animal Ethics Committee of Shanghai Normal University (Ethics Approval Number: 2024-113), while those involving minipigs were approved by the Institutional Animal Care and Use Committee (Ethics Approval Number: AMS F2408022P).

## Data availability

The data that support the findings of this study are available from the corresponding authors upon request.

## Declaration of competing interest

The authors declare the following personal relationships which may be considered as potential competing interests: Yiran Shao and Jinyu Zeng are currently employed by Shanghai Yapeng Biological Technology Co., Ltd.
