# Supplementary material for: Zinc silicate microspheres-mediated bacterial metabolic interference and microenvironment remodeling for chronic wound repair
Source: Bioact Mater. 2026 Sep 17;67:902–21. doi: 10.1016/j.bioactmat.2026.07.052 (PMC13595150; doi:10.1016/j.bioactmat.2026.07.052)
Supplement: Supplementary file 1 [file mmc1.docx]

**Supporting Information**

**Zinc Silicate Microspheres-Mediated Bacterial Metabolic Interference and Microenvironment Remodeling for Chronic Wound Repair**

**A: Experimental Section**

***Materials preparation***

**Preparation of experimental group:** Flower-like zinc silicate (Zn_2_SiO_4_, ZnSi) porous microspheres (noted as ZnSi FPMPs) were synthesized using TEOS (Si(OC_2_H_5_)_4_, AR, Sinopharm Chemical Reagent Co., Ltd., Shanghai, China) and Zn(NO_3_)_2_·6H_2_O (AR, Sinopharm Chemical Reagent Co., Ltd, Shanghai, China) as sources of Si and Zn, CTAB(C_19_H_42_BrN, AR, Aladdin, Shanghai, China) was used as surfactant.

In a typical synthesis, 0.3g CTAB was dissolved in 30 mL deionized water under magnetic stirring, after being dissolved, 0.319 mL TEOS was added with 30 min stirring. When TEOS was completely hydrolyzed, 0.595g Zn(NO_3_)_2_ and 1 mL HNO_3_ were sequentially added into the above solution drop by drop in every 30-minute interval. Later, the above solution was transferred into a Teflon-lined stainless steel autoclave and heated at 200 ℃ for 24h. The source of Zn and Si, the content of CTAB, reaction temperature and time were changed to investigate the mechanism of synthesis of ZnSi FPMPs, and the conditions were shown in Table S1. After that, the product was collected and washed with deionized water and anhydrous ethanol 3 times. Finally, the samples were calcinated at 600℃ for 4h to obtain the product.

**Preparation of control groups:** 45S5 bioglass® (98.0%, Aladdin, China) and silver sulfadiazine® powders (AgSD, Hunan Erkang Pharmaceutical Co., Ltd) were used as control groups *in vitro* antibacterial experiments and *in vivo* wound healing experiments. ZnSi FPMPs evenly spread on a 24-well polycarbonate insert (noted as T-ZnSi) was used as a control in the mechanism investigation of antibacterial property and cytocompatibility of ZnSi FPMPs.

***Materials characterization***

The phase composition of ZnSi FPMPs were examined by X-ray diffraction (XRD, D/MAX-RBX, Rigaku, Japan). Morphologies of samples were observed by a tungsten filament scanning electron microscope (FlexSEM 1000, HITACHI, Japan) and a field emission transmission electron microscope (FETEM, JEM-2100F, JEOL, Japan). Energy-dispersive spectroscopy (EDS) and selected area electron diffraction (SAED) pattern were performed to confirm the phase composition during the TEM observations. X-ray photoelectron spectrometer (XPS, Thermo Scientific K-Alpha, USA) was used to measure elements valence. BET specific surface area was tested by a surface area analyzer (Tristar II 3020, Micromeritics, USA). Particle size distribution was quantitatively measured with ImageJ sofeware.

***In vitro* degradation behavior measurement**

Phosphate buffer saline (PBS) was used to investigate the *in vitro* degradation behavior of ZnSi FMPs. Specifically, ZnSi FMPs were immersed in PBS at 37°C with the ratio of 30 mg per 10 mL, followed by immersion for 1, 4, 7, 14 and 21 days. PBS was refreshed every 3 days. After soaking, the surfaces morphology of samples was detected using a tungsten filament scanning electron microscope (FlexSEM 1000, HITACHI, Japan), the concentrations of Zn and Si of supernatant were detected by an inductively coupled plasma optical emission spectrometer (ICP-OES, Vista MPX, Varian, Palo Alto, CA, USA). For pH variation, ZnSi FMPs were immersed in PBS at 37°C with the ratio of 30 mg per 10 mL, and the pH value was recorded with a pH meter.

***In vitro a*ntibacterial capacity evaluation**

Gram-positive *Staphylococcus aureus* (*S. aureus*, ATCC 25923) and gram-negative *Escherichia coli* (*E.coli*, ATCC 25922) were selected as the representative bacterias to test the antibacterial properties, ZnSi FPMPs with different mass were used as experimental groups (1, 3, 5 mg ZnSi FPMPs, abbreviated as ZnSi-1, ZnSi-3 and ZnSi-5). ZnSi-1, ZnSi-3 and ZnSi-5 were sterilized and evenly spread at the bottom of a 24-well plate. The bacteria suspension was diluted to 1×10^7^ Colony-Forming Unit (CFU) per mL using sterilized Phosphate Buffered Saline (PBS). The absorbance of bacterial suspension was tested respectively using the cell counting kit-8 (CCK-8, MCE, HY-K0301) at 450 nm wavelengths after 24 h of culturing. Commercial products 45S5 and AgSD were used as control groups. To simulate the antibacterial properties of samples on normal skin and wound skin, 0.01M PBS with pH = 6.0, pH = 7.4 and pH = 8.5 were used as culture medium. For the antibacterial experiment, 3mg of AgSD, 45S5 and ZnSi were sterilized and evenly spread at the bottom of a 24-well plate. The bacteria suspension was diluted to 10^6^ CFU per mL using PBS with different pH values. Each sample was cultured with 1 mL bacterial suspension and incubated at 37 ℃ for 24 h, and four specimens of each group were tested. After culturing, the absorbance of the bacterial suspension was tested, respectively, using CCK-8 assay. Moreover, the bacterial suspension after culturing was diluted and cultured on Tryptic Soy Broth (TSB)/ Luria-Bertani (LB) agar plate at 37 °C for 16 h, followed by photography. For SEM (FlexSEM 1000, HITACHI, Japan) observation, the cultured bacteria were fixed with 2.5% glutaraldehyde, and then dehydrated with a series of ethanol solutions (30, 50, 75, 90, 95 and 100 v/v%).

Morphologies of *S. aureus* after culturing with the Ctrl and ZnSi groups were observed by a transmission electron microscopy (TEM, HT7800, Hitachi, Tokyo, Japan).Quantitative colony counting (log_10_ (CFU/mL)), minimum inhibitory concentration (MIC, μg/mL) and minimum bactericidal concentration (MBC, μg/mL) of ZnSi against *S. aureus* at pH 6.0, 7.4 and 8.5 were further determined to measure the pH-independent antibacterial property of ZnSi FPMPs. For colony counting, the bacterial suspension after culturing was diluted and cultured on TSB agar plate at 37 °C for 16 h, and the bacterial CFU were counted. The bacterial concentration was calculated using the equation: Bacterial concentration (CFU/mL) = (number of colonies × dilution factor)/ volume of inoculum (mL). The MIC and MBC of ZnSi FMPs against *S. aureus* under different pH conditions were determined via the standard microbroth dilution method in 96-well plates, rigidly adhering to the clinical guidelines established by the Clinical and Laboratory Standards Institute (CLSI M07 and CLSI M26). Briefly, the ZnSi FMPs were subjected to 2-fold serial dilutions at designated pH values (6.0, 7.4, and 8.5) to achieve a concentration range from 8 to 1024 $\mu\text{g/mL}$. Each well was then inoculated with a standardized bacterial suspension to yield a final density of approximately 5×10^5^ CFU/mL. After incubation at 37 ℃ for 24 h, the MIC was defined as the lowest concentration of ZnSi FMPs that completely inhibited visible bacterial growth. To subsequently determine the MBC, a $10 \mu\text{L}$ suspension from each clarified well showing no visible growth was cultured onto TSB agar plates. Following an additional 24 h of incubation, the MBC was recorded as the lowest material concentration that eradicated $\geq99.9\%$ of the initial bacterial inoculum.

To assess the antibacterial duration, the antibacterial capacity of ZnSi FPMPs after degradation was also performed, and 45S5 was used as a control. Specifically, the samples were immersed in PBS (pH = 7.4) with the ratio of 3 mg/ mL, and incubated for 1, 4 and 7 days, respectively, at 37 ℃ in a shaker. PBS was exchanged every 2 days during immersion. At each time point, the powders were collected by centrifugation. Finally, the antibacterial experiment procedure of samples after degradation for different times was consistent with the previous steps, using PBS with pH = 7.4.

***Mechanism investigation of the antibacterial property of ZnSi FPMPs***

After the comprehensive antibacterial test of ZnSi FPMPs, RNA sequencing (Shanghai Personalbio Biotechnology Co., Ltd) was performed to investigate the mechanism of antibacterial property of ZnSi FPMPs, the Ctrl and ZnSi FPMPs evenly spread on 24-well polycarbonate insert (noted as T-ZnSi) was used as a control group. The blank control (Ctrl), T-ZnSi and ZnSi FPMPs were placed in a 24-well plate with 3 replicate samples in each group, and 1 mL of *S. aureus* bacterial suspension (5 × 10^7^ CFU/mL) was added to each well. After culturing for 6 h at 37 ℃. Afterwards, the supernatant was collected to analyze the concentration of Zn using an inductively coupled plasma optical emission spectrometer (ICP-OES, Vista MPX, Varian, Palo Alto, CA, USA). The total bacterial RNA was extracted to conduct RNA sequencing, and the data was analyzed via the online platform Personalbio GenesCloud (https://www.genescloud.cn), while the differentially expressed genes of bacterial between Ctrl, T-ZnSi and ZnSi groups were compared. Moreover, the expression of *ilvA*, *ilvC* and *leuC* genes in *Valine, leucine and isoleucine biosynthesis* pathway, *rpsG* and *rpsM* genes in *Ribosome* pathway were determined. The sequences of the PCR primers were listed in Table S2, and *gyrB* served as the housekeeping gene.

***In vitro angiogenesis and anti-inflammatory assessment of ZnSi FPMPs***

Human umbilical vein endothelial cells (HUVECs) and mouse macrophage cells (RAW 264.7) were used to test the effect of ZnSi FPMPs on angiogenesis and anti-inflammation. The cells were cultured with α-MEM, supplemented 10% FBS and 1% antimicrobials of penicillin and streptomycin in a 5% CO_2_ incubator at 37°C.

**Cytocompatibility test**: The cell counting kit (CCK-8, Dojindo, Kumamoto, Japan) assay was used to evaluate the cytocompatibility of samples. The cells were seeded into 24/96-well plates and cultured for 24h. Hence, the culture media were replaced by different concentrations of ZnSi/T-ZnSi suspensions. At each timepoint, the absorbance of the culture medium was measured at wavelengths of 450 nm. Besides, live-dead staining assay was also performed to evaluate the cell viability of different concentrations of ZnSi FPMPs using a staining kit (Invitrogen^TM^, Thermo Scientific, US, R37601), and the stained cells were observed on a confocal laser microscope (Leica DM5500, Leica Microsystems, Germany). Moreover, immunofluorescent staining of TNF-α (A11534, ABclonal) of Raw 264.7 was captured on day 2 to evaluate the anti-inflammatory effect of ZnSi FPMPs.

**Cell migration and tube formation**: HUVECs were seeded in a 6-well plate with 4×10⁵ /well, and a linear scratch was created using a 10 μL pipette tip after 90 % confluence was achieved. Hence, cells were cultured with ZnSi FPMPs, and the scratch closure was monitored at 0 and 12 h using an inverted optical microscope. Images were quantified with ImageJ software. For tube formation, 200 μL per well of Cultrex® Reduced Growth Factor Basement Membrane Extract (Cultrex®BME, Trevigen, Gaithersburg, MD, USA) was first distributed in 48-well plates and pre-cultured for 30 min. Subsequently, 500 μL of HUVECs (5 ×10^4^ cells) pre-cultured with ZnSi medium for 3 days were seeded on the prepared Matrigel. After 8 hours of continuous culture, tube formation images were captured with a light microscope, and the number of junctions, meshes and branches was counted with ImageJ software.

**Flow cytometry analysis of RAW 264.7 polarization:** The RAW 264.7 cell line was treated with ZnSi medium supplemented with LPS (500 ng/mL) for 2 days. Then, cells were immunophenotyped using fluorescent-conjugated antibodies: FITC-anti-CD11b (Biolegend, 101208, monocyte marker), PE-anti-F4/80 (Biolegend, 123108, macrophage marker), PE-Cy7-anti-CD86 (Invitrogen, 2524242, M1 marker), and AF647-anti-CD206 (Invitrogen, 2611851, M2 marker). Data acquisition was conducted on a CytoFLEX flow cytometer (Beckman). The mean fluorescence intensity (MFI) of CD86, or CD206, and the ratio of CD206/CD86 were also quantified using FlowJo 10.9 software.

**Angiogenic and anti-inflammatory gene expression:** Quantitative real-time polymerase chain reaction (qRT-PCR) was conducted to quantify the angiogenic and anti-inflammatory activity of ZnSi. Briefly, 2 mL of HUVECs/Raw264.7 (5 × 10^4^ cells/mL) suspension was seeded in 6-well plates per well and cultured for 24h. Subsequently, cell culture medium was replaced by ZnSi medium, respectively. After 2 days stimulation, total RNA was collected and then converted into cDNA. Hence, the expression of vascular-related genes KDR, HIF-α, CD31, VEGF-α, FGF2 and VWF, M1 macrophage markers TNF-α, iNOS and CD86, and M2 macrophage markers IL-4, IL-10 and CD206 were determined. The sequences of the PCR primers were listed in Table S2, and GAPDH served as the housekeeping gene.

***In vivo assessment of ZnSi FPMPs on S. aureus-infected wounds in mice***

*S. aureus*-infected wounds in mice were established to assess the *in vivo* antibacterial capacity and healing promotion of ZnSi FPMPs. To assess the ability of ZnSi FPMPs on infected wound, commercial AgSD and 45S5 were used as control groups (3 mg AgSD and 45S5, abbreviated AgSD-3 and 45S5-3) and different mass of ZnSi FPMPs (ZnSi-1, ZnSi-3 and ZnSi-5) were also chosen to analyze their optimal content. This animal experiment was performed at the Shanghai Normal University. Eight-week-old male ICR mice were purchased from Shanghai Reagan Technology Co., Ltd. To create the infected wound model, two 6 mm full-thickness wounds were firstly established on the back of each mouse and 20 uL of S. aureus bacterial suspension (1 × 10^8^ CFU/mL) was added to each wound, followed by infection for 24 h according to the literature. Later, the infected wound mice were randomly classified into six groups, the blank control group (Ctrl), 3 mg AgSD (AgSD-3), 3 mg 45S5 (45S5-3), 1 mg ZnSi (ZnSi-1), 3 mg ZnSi (ZnSi-3) and 5 mg ZnSi (ZnSi-5). 5 mice for each group. Macroscopic wound photos at days 0, 1, 5, 7, 9 and 12 were analyzed via the Image J software (NIH, USA) to calculate wound closure rate. Also, the wound exudate was collected at days 0, 1, 5 and 7 to evaluate the antibacterial capacity of samples, and the bacterial activity were analyzed through OD_450_ values and cultivation on TSB agar plate.

On the 12^th^ day, all the animals were sacrificed with an overdose of non-diluted pentobarbital and the wound tissue were fixed in 4% paraformaldehyde for 24 h, followed by embedding, cutting into slices and staining analysis including hematoxylin and eosin (HE), Masson trichrome staining, and immunofluorescence staining for CD68/CD163(inflammation markers) and CD31/α-SMA (vascular markers).

***Mechanism investigation of in vivo healing promotion of ZnSi FPMPs***

RNA sequencing was performed to explore the mechanism of *in vivo* healing promotion of ZnSi FPMPs. 6 mm full-thickness wound tissue of blank control and ZnSi treated groups were collected on 7 days for RNA sequencing (Shanghai Qianya Kangyao Biotechnology Co., Ltd). Each group contained two parallel samples, while each sample contained four wound tissues. The differentially expressed genes of wound tissues between the Ctrl and ZnSi groups were compared.

***In vivo assessment of ZnSi on diabetic wounds in mice***

Diabetic wounds were established to assess the *in vivo* anti-inflammatory and healing promotion of ZnSi FPMPs on chronic wounds healing, and commercial AgSD and 45S5 were used as control groups. This animal experiment was performed at the Shanghai Normal University. Eight-week-old male ICR mice were purchased from Shanghai Reagan Technology Co., Ltd. A diabetic wound model was established with 1% streptozotocin chain (STZ) intraperitoneal injection, until the blood glucose value was greater than 15 mg/mL and stabilized for 10 days. Subsequently, two 6 mm full-thickness wounds were established on the back of mice, and the diabetic wound mice were randomly classified into four groups with 5 mice for each group, Ctrl, AgSD, 45S5 and ZnSi, and the content of samples on each wound was 3 mg. Macroscopic wound photos on days 0, 1, 5, 9 and 12 were recorded to calculate wound closure rate. On the 12^th^ day, all the animals were sacrificed, and all wound tissues were collected. The subsequent histological analyses were consistent with the previous steps.

***In vivo assessment of ZnSi FPMPs on S. aureus-infected wound in minipigs***

*S. aureus*-infected wounds in minipigs were also established to assess the healing effect of ZnSi FPMPs, and commercial AgSD and 45S5 were also used as control groups. This animal experiment was performed at the Shenzhen Advanced Animal Study Service Center. Guangxi Bama mini-pigs (weight 25-30 kg) were obtained to create a 1.0 × 2.0 cm^2^ full-thickness *S. aureus* infected wound model. Subsequently, the 16 infected wound minipigs were randomly classified into four groups: the blank control group (Ctrl), 20 mg AgSD, 45S5 and ZnSi FPMPs. One pig for each time point and four parallel wounds for each group. Macroscopic wound photos were recorded on Days 0, 3, 10 and 28 and the wound area was calculated using Image J software (NIH, USA). The skin wound tissue was collected to isolate residual bacteria on days 3 and 10, and the bacterial activity were evaluated through bacteria cultivation on TSB agar plates.

The skin wound tissue was fixed in 4% paraformaldehyde for 48 h, followed by embedding, cutting into slices, and staining analysis including hematoxylin and eosin (HE), Masson trichrome staining. Inflammation was evaluated via an immunofluorescence staining of IL-6 (Ab9324, Abcam) and CD206 (Ab64693, Abcam). Blood vessels were assessed by CD31 (AF3628, R&D Systems) and α-SMA (MA5-11547, Thermo-Fisher) staining.

***Statistical analysis***

All quantitative data *in vitro* and *in vivo* were from three or more than three parallel samples. Statistical analysis was performed by GraphPad Prism 8.0 (GraphPad) using one-way or two-way analysis of variance (ANOVA) or Student’s t-test.

**B: Supplementary figures**

Table S1. Conditions of zinc silicate synthesis

| No. | Source of Zn | Source of Si | T (℃) | CTAB (g) | t (h) |
| --- | --- | --- | --- | --- | --- |
| 1-1 | ZnF_2_ | TEOS | 200 | 0.3 | 24 |
| 1-2 | ZnCl_2_ | TEOS | 200 | 0.3 | 24 |
| 1-3 | ZnSO_4_ | TEOS | 200 | 0.3 | 24 |
| 1-4 | Zn(CH_3_COO)_2_ | TEOS | 200 | 0.3 | 24 |
| 1-5 | Zn(NO_3_)_2_ | Na_2_SiO_3_ | 200 | 0.3 | 24 |
| 1-6 | Zn(NO_3_)_2_ | TEOS | 200 | 0.3 | 24 |
| 2-1 | Zn(NO_3_)_2_ | TEOS | 200 | 0 | 24 |
| 2-2 | Zn(NO_3_)_2_ | TEOS | 200 | 0.05 | 24 |
| 2-3 | Zn(NO_3_)_2_ | TEOS | 200 | 0.1 | 24 |
| 2-4 | Zn(NO_3_)_2_ | TEOS | 200 | 0.2 | 24 |
| 2-5 | Zn(NO_3_)_2_ | TEOS | 200 | 0.5 | 24 |
| 3-1 | Zn(NO_3_)_2_ | TEOS | 200 | 0.3 | 6 |
| 3-2 | Zn(NO_3_)_2_ | TEOS | 200 | 0.3 | 8 |
| 3-3 | Zn(NO_3_)_2_ | TEOS | 200 | 0.3 | 12 |
| 4-1 | Zn(NO_3_)_2_ | TEOS | 120 | 0.3 | 24 |
| 4-2 | Zn(NO_3_)_2_ | TEOS | 150 | 0.3 | 24 |

**Table S2.** PCR primer sequences

| Genes | Forward sequences (5’-3’) | Reverse sequences (5’-3’) |
| --- | --- | --- |
| *KDR* | GGAACCTCACTATCCGCAGAGT | CCAAGTTCGTCTTTTCCTGGGC |
| *VEGFA* | TTGCCTTGCTGCTCTACCTCCA | GATGGCAGTAGCTGCGCTGATA |
| *HIF1A* | TATGAGCCAGAAGAACTTTTAGGC | CACCTCTTTTGGCAAGCATCCTG |
| *CD31* | AAGTGGAGTCCAGCCGCATATC | ATGGAGCAGGACAGGTTCAGTC |
| *FGF2* | AGCGGCTGTACTGCAAAAACGG | CCTTTGATAGACACAACTCCTCTC |
| *VWF* | CCTTGAATCCCAGTGACCCTGA | GGTTCCGAGATGTCCTCCACAT |
| *INOS* | GAGACAGGGAAGTCTGAAGCAC | CCAGCAGTAGTTGCTCCTCTTC |
| *TNFA* | GGTGCCTATGTCTCAGCCTCTT | GCCATAGAACTGATGAGAGGGAG |
| *CD86* | ACGTATTGGAAGGAGATTACAGCT | TCTGTCAGCGTTACTATCCCGC |
| *IL4* | ATCATCGGCATTTTGAACGAGGTC | ACCTTGGAAGCCCTACAGACGA |
| *IL10* | CGGGAAGACAATAACTGCACCC | CGGTTAGCAGTATGTTGTCCAGC |
| *CD206* | GTTCACCTGGAGTGATGGTTCTC | AGGACATGCCAGGGTCACCTTT |
| *GAPDH* | GTCTCCTCTGACTTCAACAGCG | ACCACCCTGTTGCTGTAGCCAA |
| *ilvA* | CGTTGAAGTTGTACTCACTGGTGAT | TCTAGCATTTCTTTAGCAAGCGTAC |
| *ilvC* | AAGGTTCTGCTGTACCATCACTATT | ACCGCAAAGTACTGCTTGTTCA |
| *leuC* | TGGGCGAACCGCAACTATTA | TCAGGTCCTACCATGTGAACAATAC |
| *rpsG* | CGTCCAGAGCGTCGTACTACTTT | TTCTTAACGGCACCACCTGTATT |
| *rpsM* | AACTTCACGGATGCGACCTA | TATTGCAGGAGTAGATATTCCACGT |
| *gyrB* | ATGACTGATGCCGATGTGGATG | GCCTGCTTCAATTAACGGTCTC |

Figure S1. Schematic models and SEM images of ZnSi FMPs synthesized under different conditions, (A) Raw materials, (B, C) CTAB content and (D, E) Reaction time and temperatures.

Figure S2. (A) pore size distribution and (B) pore volume of ZnSi FMPs synthesized under different content of CTAB.

Figure S3. Degradation behavior of ZnSi FMPs. (A) SEM morphology of ZnSi FMPs, (B) pH value, Concentration of (C) Zn ion and (D) Si ion of PBS after immersion for different days (n=3).

Figure S4. Absorbance results of *S. aureus* cultured with Ctrl, 45S5 and ZnSi groups after 1D, 4D and 7D degradation (n=3). ** *p <* 0.01, *** *p <* 0.001, by two-way ANOVA with Bonferroni *post hoc* test.

Figure S5. Concentration of Zn ion in PBS after cultured with *S. aureus*, *S. aureus* + T-ZnSi, *S. aureus* + ZnSi(n=3). **** *p <* 0.0001, by one-way ANOVA with Bonferroni *post hoc* test.

Figure S6. Heatmap of the top up-regulated/down-regulated DEGs (A) GO enrichment analyses of DEGs of T-ZnSi *vs* Ctrl, ZnSi *vs* Ctrl and ZnSi *vs* T-ZnSi and (B) KEGG enrichment analyses of DEGs of T-ZnSi *vs* Ctrl, ZnSi *vs* Ctrl and ZnSi *vs* T-ZnSi.

Figure S7. (A) GO enrichment analyses of down-regulated DEGs of T-ZnSi *vs* Ctrl, ZnSi *vs* Ctrl and ZnSi *vs* T-ZnSi. (B) GO enrichment analyses and (C) KEGG enrichment analyses of up-regulated DEGs of T-ZnSi *vs* Ctrl, ZnSi *vs* Ctrl and ZnSi *vs* T-ZnSi.

Figure S8. Heatmap of DEGs involved in (A) *Pyrimidine metabolism pathway and* (B) *Galactose metabolism* of three groups (Ctrl, T-ZnSi, ZnSi).

Figure S9. (A) Cytotoxicity results of RAW 264.7 cultured with different ZnSi content and (B) Cell proliferation assays of RAW 264.7 cultured with ZnSi (n=4). * *p <* 0.05, *** *p <* 0.001, by two-way ANOVA with Bonferroni *post hoc* test.

Figure S10. HE staining of heart, liver, spleen, lung, and kidney of mice treated with Ctrl, AgSD, 45S5, ZnSi-1, ZnSi-3 and ZnSi-5 for 12 days.
